# Characterizing multidimensional facial synchrony for relationship quality detection

**DOI:** 10.1371/journal.pone.0356071

**Published:** 2026-10-01

**Authors:** Malak Fora, Alyssa Miville, Richard Mattson, Congyu Wu

**Affiliations:** 1 School of Systems Science and Industrial Engineering, Binghamton University, New York, United States of America; 2 Department of Psychology, Binghamton University, New York, United States of America; Leuphana University of Lüneburg: Leuphana Universitat Luneburg, GERMANY

## Abstract

Relationship quality is typically assessed using self-report questionnaires, which are time-consuming and may not fully capture interpersonal processes during interaction. Automated facial expression analysis provides an opportunity to study nonverbal behavior during naturalistic interactions in a more objective and continuous manner. We characterize dyadic facial synchrony from video-based facial action unit time series and examine its association with marital relationship quality. Specifically, dyadic coordination is quantified using zero-lag cross-correlations between partners’ facial behaviors across multiple action units. Computed over successive temporal windows, these synchrony measures form a multidimensional synchrony profile, termed the S-matrix. We evaluate the proposed method using an iMotions dataset comprising video recordings of in-person conversations, frame-level facial expression estimates, and Quality of Marriage Index (QMI) survey responses from 27 married couples. Results from an unsupervised clustering analysis demonstrate that the proposed synchrony representation provides better separation between couples with high and low marital relationship quality than conventional statistical feature baselines.

## 1 Introduction

Assessment of relationship quality is critical to understand the social dynamics and wellbeing of married couples. Traditional assessment tools, which are predominantly survey inventories, can be burdensome and susceptible to biases when not administered properly. Recent advancements in computer vision have enabled real-time facial expression recognition (FER) of individuals engaging in face-to-face communication. These facial expressions are nonverbal communication cues shown to reflect emotional connection and relationship quality. As such, it may be feasible to use automated facial expression data produced by video cameras and emotion recognition software to detect different levels of relationship quality. If successful, this approach can serve to streamline and objectify relationship quality assessment.

Research shows that *facial synchrony*, defined as the degree of coordination of facial movements between two people during interaction [1], correlates positively with empathy, rapport, and relationship satisfaction [2,3]. Therefore, precise characterization of facial synchrony can be key to predicting relationship quality. Existing attempts at characterizing facial synchrony using FER data involve manually tallying and comparing total moments of positive and negative effects experienced by both partners during a recorded episode of interaction [4]. Other methods for example the correlation and cross-lag correlation [5] utilize a cross-time series approach which typically aggregates the dynamics of an entire session to a single synchrony score, thus failing to capture moment-to-moment variations during interaction.

According to Social Baseline Theory [6], human brains are ‘hardwired’ to rely on social proximity, which is maintained through a relational attunement between dyad members. Facial expressions serve as a primary conduit for this process, functioning as high-bandwidth signals of internal affective states and interpersonal intentions that facilitate the real-time coordination of social interaction (see Bibby et al. [7]). Therein, synchrony serves as a physical manifestation of shared or intersubjective psychological space, wherein partners coregulate emotional states and construct meaning. In a marital context, high-quality relationships might be characterized by higher degrees of attunement. While aggregate measures of facial synchrony provide a snapshot of interpersonal connection, they fail to capture the temporal fluidity essential to modern theories of dyadic co-regulation. Relationship quality is not merely a product of total shared affect, but of the dyad’s ability to dynamically shift synchrony in response to evolving conversational, contextual, and relational demands. Moreover, by utilizing a multidimensional approach that incorporates a range of facial affects (e.g., joy, contempt, neutral), the current study moves beyond simple valence to capture the complexity of this shared interpersonal space. Our approach uses a sliding window to calculate a cross-correlation for each facial action unit to construct a synchrony profile for each dyad. This profile, which we term the *S* -matrix, represents the dynamic state of the dyad during interaction. We used the data recorded for married couples to study their quality of relationship. By applying an unsupervised pipeline, we categorize the quality of the relationship as either stressed or relaxed based on these synchrony profiles. We found that facial synchrony, as measured by facial action units, can effectively differentiate between married couples with total low and high Quality of Marriage Index (QMI) scores. This work advances prior research by introducing a fully automated, unsupervised framework for characterizing marital interaction dynamics based on time-varying facial synchrony, rather than relying on aggregate or static measures.

## 2 Related work

### 2.1 Marital relationship quality

Measures of marital quality, such as QMI used in this study, capture an individual’s overall subjective evaluation of their relationship, which is essential for predicting health and relational outcomes. Lower levels of quality are typically linked to higher levels of depressive symptoms [8], greater feelings of loneliness [9], and broader physical and mental health challenges [10]. Such measures are also arguably more informative than assessments of marital stability (e.g., divorce), because they allow researchers to differentiate between more or less well-functioning couples by degree, as well as to track changes in how individuals feel about their relationship over time. They are also the common metric by which marital researchers evaluate the empirical relevance of other variables and processes theorized to be important to relationship functioning.

How partners *communicate* with each other is an important determinant of marital quality. For instance, negative premarital communication patterns (e.g., problem denial) are often associated with high levels of discord, distress, and divorce [11]; whereas effective and attentive communication styles between partners are related to higher marital satisfaction and greater stability longitudinally [12]. Research supports the notion that observations of communication behaviors between partners can elicit data that is able to discriminate between happy and unhappy couples. Importantly, the communication of emotion (e.g., joy versus anger), often via facial expressions, has been recognized as a key component of dyadic interaction [13]. Researchers have suggested that facial expressions are predictive of significant marital outcomes [14], and deficits in the recognition of such expressions are associated with lower relationship satisfaction [15]. As such, we are motivated in this study to utilize automated measurements of facial expressions during dyadic communication between married partners to detect marital relationship quality.

### 2.2 Automated measurements of facial expressions

Facial expressions provide rich evidence for understanding emotional responses and interaction dynamics. Therefore, their automatic identification has been a prominent area of research in computer science and can enable various emotion-aware human-computer interaction applications in social robotics, user experience, security, and healthcare.

Videos and muscle movements of the face are two main data sources researchers utilize to achieve this task. Facial muscle movement signals, captured by facial electromyography (fEMG), are capable of detecting subtle electric activity and visually unobservable muscle movements [16]. However, due to the necessity of attaching electrodes to the face, fEMG is unsuitable for most real world data collections outside the lab. Video data, on the other hand, allows more discrete data collection with minimal to no burden on the participants. Videos of facial expressions are then codified into time series of facial movement readings for further analysis. An established protocol for video-based facial movement coding is the Facial Action Coding System (FACS) which was developed by Paul Ekman and Wallace Friesen [17] that describes facial expressions using action units. Each action unit corresponds to the visible motion or positioning of an individual muscle or muscle group (e.g., “AU1: inner brow raise”). Combinations of action units can further be mapped to facial affects; for example, *joy* can be represented by the simultaneous presence of AU6 (cheek raiser) and AU12 (lip corner puller).

Conventional facial movement coding is done manually by a human observer which is time and resource consuming thus impractical for producing real-time or large quantities of data. With advancements in computer vision and artificial intelligence [18,19], facial expression recognition software can now read in videos as input and track facial action units frame by frame to generate annotations of facial movements and facial affects automatically. Automated facial expression data facilitates more temporally granular characterizations of communication dynamics and thus is what we use as input data for interactions between married partners in this study.

### 2.3 Characterization of facial synchrony

An established but somewhat simplistic way to assess facial synchrony involves counting labeled instances of negative or positive emotions exhibited by the interlocutors [4,20–22]. This method quantifies the similarity between the two interlocutors’ emotional experiences during interaction, but overlooks the temporal dependency between consecutive moments. To characterize such temporal dependency, multiple cross time-series methods have been proposed to compute synchrony scores using facial action unit time series data, however, each of these methods quantifies facial synchrony on an aggregated level, producing one synchrony score as output based on two complete time series of facial action units [5,23]. Such a “global” synchrony score reveals an overall level of facial coordination during dyadic interaction [24] but does not describe the variation within a session.

Facial coordination can be characterized along two independent dimensions: (i) temporal resolution and (ii) coupling form. With respect to coupling form, synchrony is often conceptualized as same-time similarity (zero-lag alignment). Yet coordination may also take the form of time-lagged, or complementary patterns, where one partner’s facial activity systematically precedes, follows, or contrasts with the other’s (e.g., leader–follower and response dynamics [25–27]. These lagged/complementary relationships can be quantified either as a single session-level estimate (global lag structure) or as time-varying estimates using a sliding window (moment-to-moment lag structure).

In the present work, we focus on the temporal-resolution dimension by introducing a sliding-window framework that tracks moment-to-moment changes in facial coordination throughout the interaction. Within each window, we adopt a zero-lag formulation to quantify simultaneous coupling across action units, enabling a fully automated, interpretable, and computationally efficient approach for capturing time-varying concurrent synchrony. We focus on zero-lag coupling in the present work; lagged and complementary formulations, which have been explored in prior literature, are beyond the scope of this study. Rather than studying delay or directionality, the present work adopts a systems-level perspective that emphasizes the overall structure of synchronous facial coordination, using a zero-lag formulation to describe simultaneous coupling across action units within short time windows. This design choice enables a fully automated, easy-to-interpret, and computationally efficient characterization of moment-to-moment concurrent synchrony. Despite its conceptual simplicity, the zero-lag formulation proved sufficient to reveal clear and stable separation between dyads in the present dataset, demonstrating that time-localized concurrent synchrony alone captures meaningful structure related to relationship quality. These findings suggest that moment-to-moment zero-lag synchrony constitutes a strong and informative baseline for characterizing facial coordination in dyadic interaction

## 3 Data and methods

### 3.1 Data

An archival data set collected between the years 2015 and 2017 at a mid-sized university in the Northeastern part of the United States was utilized for the purposes of this study. The study was conducted in accordance with the ethical standards of Binghamton University’s Institutional Review Board (IRB), and approval was granted under Protocol Number 3030−13. All participants provided written informed consent prior to participation.

The dataset was de-identified and provided to the research team in October 12, 2023. The dataset analyzed in this study is publicly available online via Zenodo [28]. All personal identifiers were removed prior to data access, ensuring compliance with ethical standards for human subjects research. The initial data set comprised ≈150 engaged heterosexual couples; however, only 27 couples were selected for analysis (see section 3.3 for details). Within this selected sample, partners were between 23–54 years of age. The majority of partners (85%) were White, and over half of the partners (59%) received at least a 4-year college education. These couples were recruited using various outreach methods common to marital and relationship research at that time (e.g., targeted mailings, Facebook posts, local bus and radio advertisements). Criteria for participation in this study required eligible study participants to be (a) at least 18 years old, (b) able to speak, write, and understand English, and (c) married.

When couples arrived in the laboratory, each partner reviewed and signed consent form after being informed of the purposes of this study by a laboratory assistant. The couples were then placed in separate rooms briefly to independently complete self-report measures related to their relationship and individual-level aspects relevant to marriage (e.g., health). After questionnaires were completed, partners were brought into the same room, where they were seated facing each other at a 90° angle, with separate mounted cameras directed at each partner. They were instructed to engage in two 10-minute social support discussions (e.g., with each social support discussion focused on a topic of each partner’s choosing). This type of task was selected due to research suggesting couples’ affective behavior may differ depending on which partner selected the topic [29]. Couples were thanked for their time and compensated with $100 at the conclusion of their laboratory visit.

For the purposes of this study, the couples were categorized based on their Quality of Marriage Index (QMI) scores. The QMI [29] is a well-validated six-item self-report measure of marital satisfaction. A meta analysis revealed the QMI to be a recommended choice for measuring relationship quality due to its high level of reliability across studies (with an average of α=.94) [30]. The brief inventory includes items such as: “Our marriage is strong,” and “My relationship with my partner makes me happy.” Partners answered the first five items on a seven-point Likert-style scale ranging from one (“strongly disagree”) to 7 (“strongly agree”), while the last item was answered on a 10-point scale ranging from one (“extremely low”) to 10 (“extremely high”). Higher scores indicate a greater perceived quality of marriage. The highest QMI score attainable for either partner is 45. Relationship quality was assessed at the dyadic level by summing the QMI scores of both partners. A threshold of 80 (See Section 4 for the rationale behind this threshold) was utilized to categorize couples into ‘Satisfied’ and ‘Distressed/At-Risk’ groups. From this study, 14 couples were categorized as having poorer quality marriages (total QMI score < 80), while the other 13 couples were classified as having good quality marriages (total QMI score ≥ 80).

Video recordings of all interaction tasks were uploaded into the iMotions software and analyzed using Affectiva’s automated facial expression analysis. Facial action unit activations were extracted frame by frame [31,32] at a temporal resolution of 40 ms per frame (25 Hz). Recordings wherein both partners were mistakenly present in the same frame (e.g., if couples moved closer to each other during the discussion) were split in half to allow for faces of each partner to be analyzed independently of one another. iMotions captured each partner’s face via a manually-specified bounded box, then the movement of facial features were detected and classified by their particular position and orientation. Specifically, 20 facial action units were tracked, representing specific facial muscle movements. Readers can refer to iMotions web page to see visual representation of each action unit. Classification algorithm in iMotions’ validated AFFDEX module translate combinations of action units into affective metrics such as disgust, anger, fear, sadness, surprise, and joy [33,34].

### 3.2 Overview of the proposed analysis pipeline

Fig 1 summarizes the proposed pipeline and the rationale for each major step. We begin with simultaneous video recordings of both partners and use an automated facial analysis tool to extract frame-level activation time series for each facial action unit (AU). Using action units provides an interpretable and standardized representation of nonverbal behavior that can be aligned across partners at each time point. To capture within-session changes in interaction dynamics (rather than collapsing the entire session into a single statistic), we apply a sliding window of width w moved by a fixed step size along the time series. Within each window, we compute a zero-lag cross-correlation for each action unit between partners to quantify the strength of time-local co-fluctuations; this choice emphasizes concurrent facial synchrony and yields a compact, consistent synchrony estimate per action unit and window. The resulting synchrony values are organized into an S-matrix for each dyad, where columns index AUs and rows index time windows, providing a structured representation of moment-to-moment synchrony states throughout the interaction. To compare dyads in a common space, we summarize and compare these window-level synchrony profiles by computing pairwise Euclidean distances between flattened S-matrix vectors of each dyad. We then apply multidimensional scaling (MDS) to obtain a two-dimensional embedding that facilitates visualization and structure discovery while approximately preserving the original distances. After observing an emergent pattern in the grouped data through visual analysis, we applied k-means clustering with k = 2 as an unsupervised step to identify dominant regimes in this embedding; this setting reflects the case of two broad interaction patterns consistent with the dichotomized QMI grouping (stressed vs. non-stressed) and provides an interpretable partition that can be compared to relationship quality labels without using them to fit the clusters.

**Fig 1:**
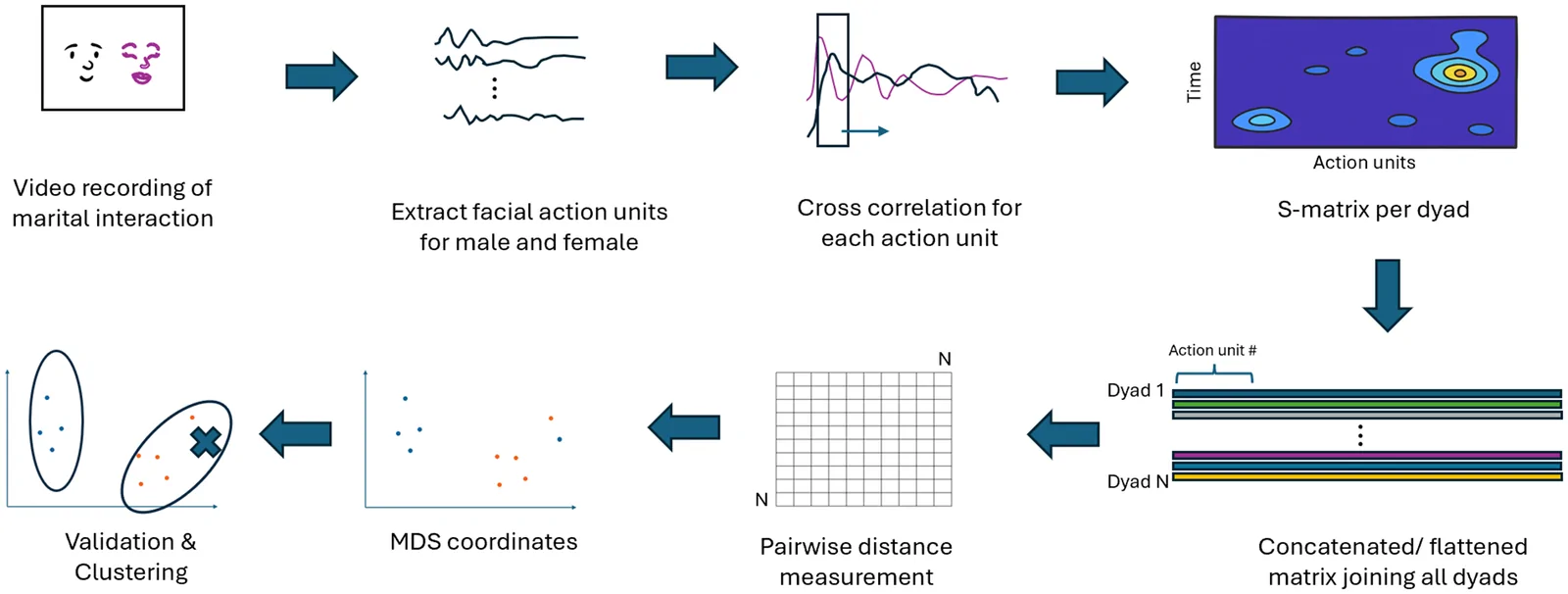
The workflow of the proposed approach for analyzing facial synchrony in dyadic marital interactions. Facial action unit time series extracted from simultaneous video recordings are analyzed using a sliding-window cross-correlation framework to construct dyad-level synchrony representations, which are subsequently embedded and clustered to examine associations with marital relationship quality.

### 3.3 Preprocessing

Several practical constraints were present in the raw dataset as the original data collection protocol was not designed for facial synchrony analysis, and the camera configuration did not ensure continuous face tracking. Consequently, differences in face visibility and tracking performance led to variability in the amount of usable data across participants and dyads. First, the male and female partners within a dyad often had different numbers of available samples, reflecting differences in recording duration and successful face tracking. Second, for some participants, action unit data were entirely missing due to unavailable video, resulting in incomplete dyads that could not be included in dyadic analyses. Third, we observed time points in which all AU values were zero simultaneously, indicating uninformative frames rather than true facial inactivity.

Data points in which all facial Action Unit values and affect codes were equal to zero were operationally treated as missing data rather than valid affective states. Within the iMotions/AFFDEX pipeline, neutral expressions are typically represented by low-magnitude, non-zero fractional values across the affect vector. In contrast, an all-zero output across the feature set is more consistent with tracking failure or missing face detection, often arising from extreme head pose or partial/full occlusion (e.g., downward gaze, hats, or out-of-frame faces).

Dyad selection was based on the availability of valid (non-zero) action unit recordings from both partners with temporal overlap (aka whether two datapoints from both participants are both available at the same timestamp). To ensure meaningful synchrony analysis, we first removed uninformative frames by excluding rows in which all action unit values were zero for a given participant. We then aligned the two partners’ time series within each dyad by retaining only timestamps for which both individuals had valid data. This ensured that synchrony measures were computed only when both partners contributed meaningful information at the same time point, which is essential for dyadic concurrent synchrony analysis. Following preprocessing, dyads exhibited variable durations of overlapping valid data, reflecting differences in recording quality, face visibility, and tracking success.

Because there was a tradeoff between maximizing the number of retained dyads and ensuring sufficient data duration for reliable synchrony estimation, a minimum threshold of 60 seconds of overlapping data was adopted. Where the first minute of available aligned data was used for subsequent analyses. This choice provided a practical balance between sample size and data quality while maintaining a uniform temporal window across dyads to capture meaningful interaction dynamics. As example, Fig 2 shows the action unit time series for the first dyad (ID: 10031004) over the selected one-minute interval. This procedure resulted in 27 retained dyads. Fig 3 illustrates the amount of available aligned data for the 27 retained dyads, highlighting differences in the number of valid frames across couples. Given the 60-s threshold, Table 1 reports participant counts at each stage of the preprocessing pipeline to document data availability and retention throughout the analysis.

**Fig 2:**
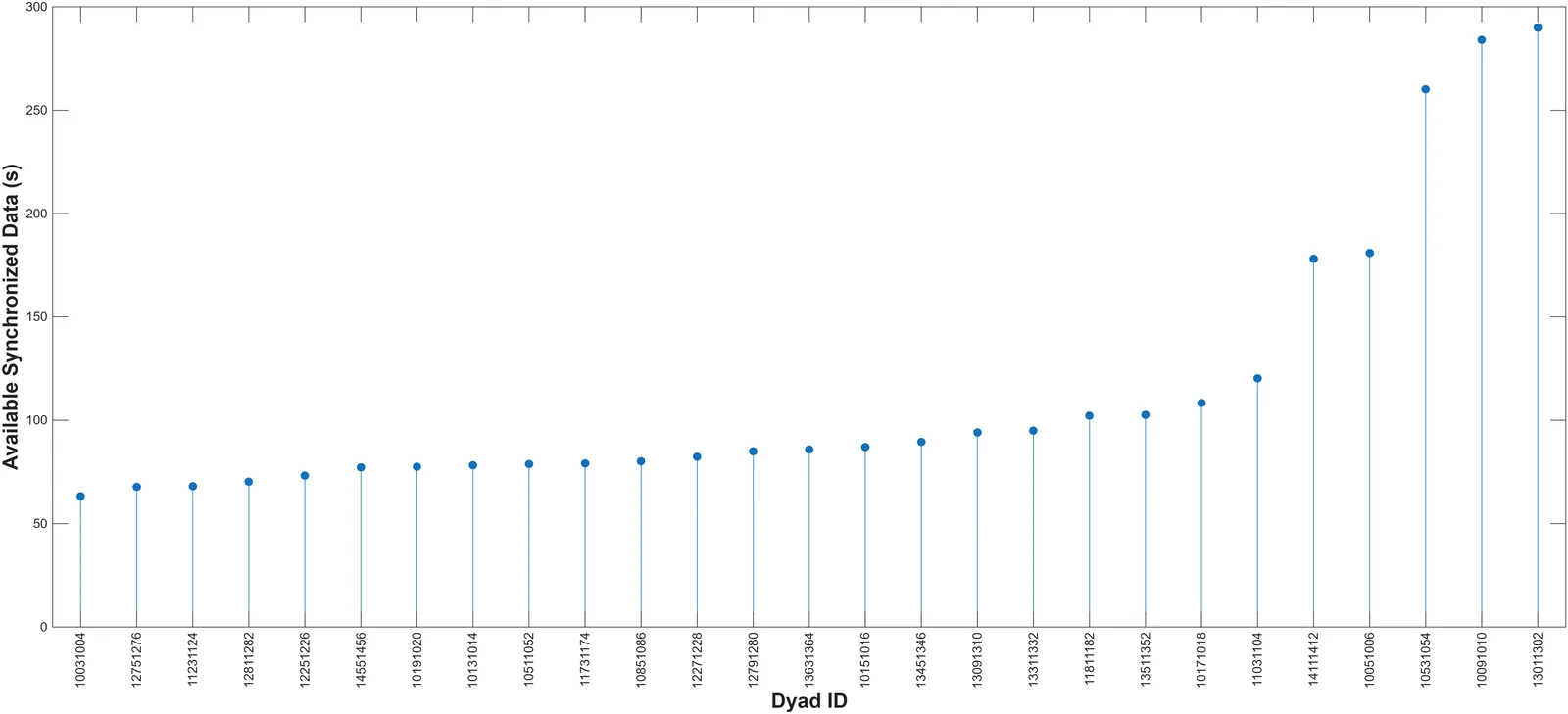
Action units of dyad (ID: 10031004) over a one-minute duration, with the husband represented in blue and the wife in red. The x-axis represents time sample, and the y-axis represents the intensity of the action unit.

**Fig 3:**
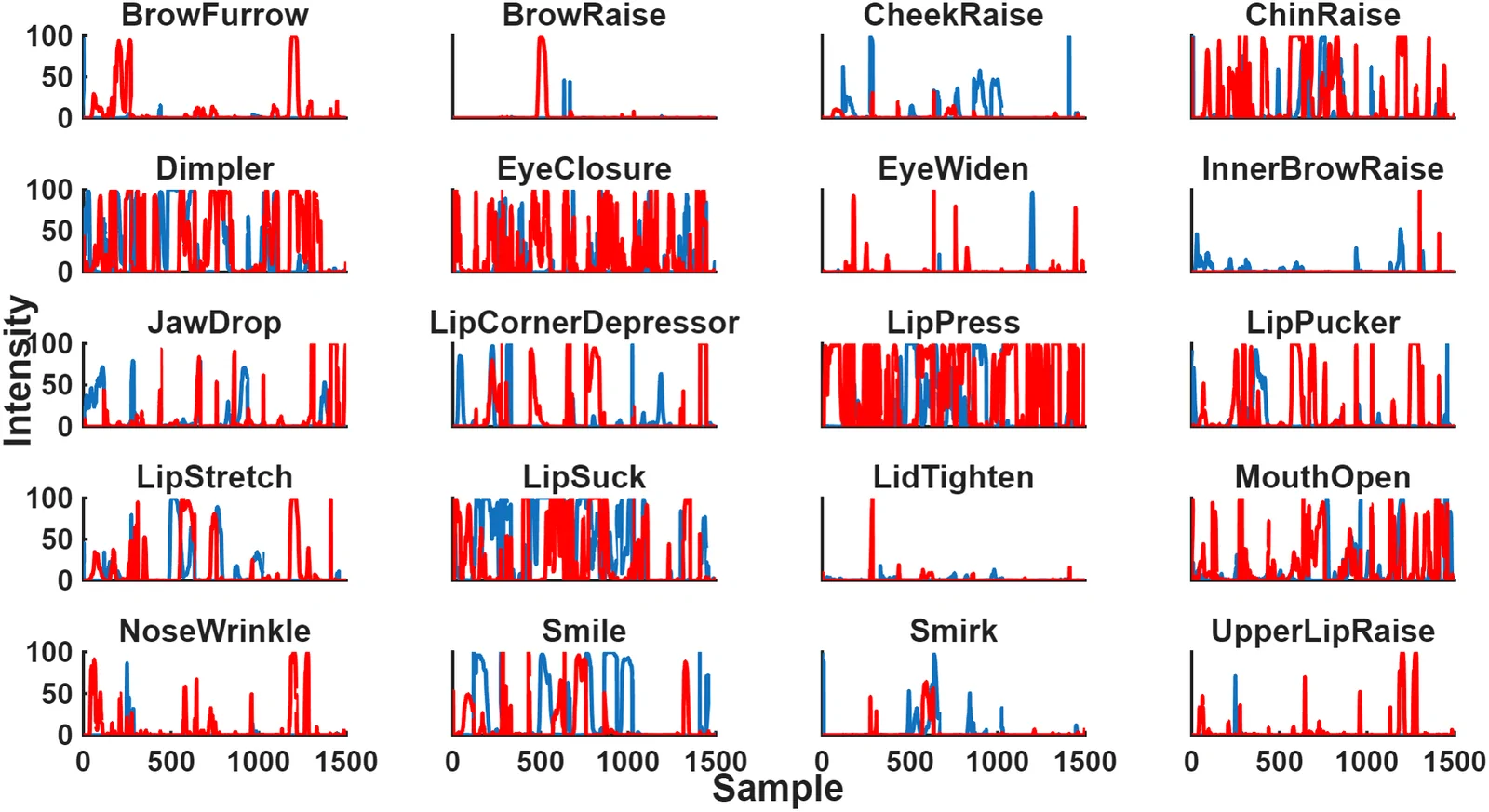
Duration of available synchronized facial action unit data for each dyad after preprocessing. The y-axis indicates the total length (in seconds) of valid, non-zero, and temporally synchronized data retained for each couple after removing uninformative frames and aligning timestamps between partners.

**Table 1:** Participant counts retained at each stage of preprocessing, considering the threshold of 60 seconds.

	Male	Female
Before filtering	120	94
After filtering	105	84
Final matched dyads	27	

### 3.4 Quantifying moment-to-moment synchrony: our proposed approach

Concurrent synchrony refers the simultaneous occurrence of coordinated behaviors, or signals between interlocutors [35]. This concept is often applied to studies of social interactions, looking at how individuals in a discussion or relationship behave at the same time. We calculated synchrony using a multidimensional windowed zero-lag cross-correlation. Given that we have 20 action units for each interlocutor in the interaction. To examine the relationship between the temporal dynamics of the interlocutors for each dimension in this state space, we performed cross-correlation analysis for each dimension individually. A sliding window of size *w* moves forward by a step size *st*. In cases where *st* is *smaller* than *w* there will be an overlap between windows and is calculated as a percentage based on Eq. 1.


$$\begin{array}{c} \text{\% Overlap}=\frac{w-st}{w}\times 100\% \end{array}$$ (1)

For each window index *i*, synchrony patterns were quantified by computing the dot product between the action unit activation time series of the two interlocutors within that window.

Formally, let


$$\begin{array}{c} {𝐗}_{i}=\left[x_{i}(1),x_{i}(2),...,x_{i}(w)\right]\;\text{and}\;{𝐘}_{i}=\left[y_{i}(1),y_{i}(2),...,y_{i}(w)\right] \end{array}$$

denote the action unit activation vectors of the two individuals within the *i*-th sliding window, where $x_{i}(j)$ and $y_{i}(j)$ represent the activation values of a given AU at the *j*-th time sample inside window *i*. The synchrony score $S_{i}$ for window *i* is then computed as Eq. 2.


$$\begin{array}{c} S_{i}=\sum_{j=1}^{w}x_{i}(j)\;y_{i}(j), \end{array}$$ (2)

where *w* denotes the window length in samples.

Various window sizes were tested with the range of 200 samples (8 seconds) and 1000 samples (40 seconds), with a fixed step size of 100 samples (4 seconds). Cheong et al. [22] employed a 30-second window for synchrony measurements, emphasizing the overall synchrony score rather than moment-to-moment variation. Fig 4 illustrates an example of synchrony measurement for the smile time series data of dyad (ID: 10031004) with (*w* = 200, *st* = 100). The figure shows each partner’s smile signal alongside the corresponding synchrony vector. In the synchrony vector, square-marked points highlight synchrony results associated with the first, eighth, and last windows out of a total of 14, as shown in the third panel, displayed over the partners’ signals. We aggregated the resulting synchrony measures from all action units into a synchrony matrix *S* -matrix. This multidimensional matrix had dimensions a×b, where *a* denotes the number of windows, and *b* is the total number of action units. *S* -matrix represents the dyad’s synchrony profile and provide a comprehensive spatiotemporal representation of facial expressions over time. Fig 5 presents contour plots of the synchrony profiles across all dyads (*w* = 200, *st* = 100). The two panels represent dyad groups defined by high versus low overall QMI scores.

**Fig 4:**
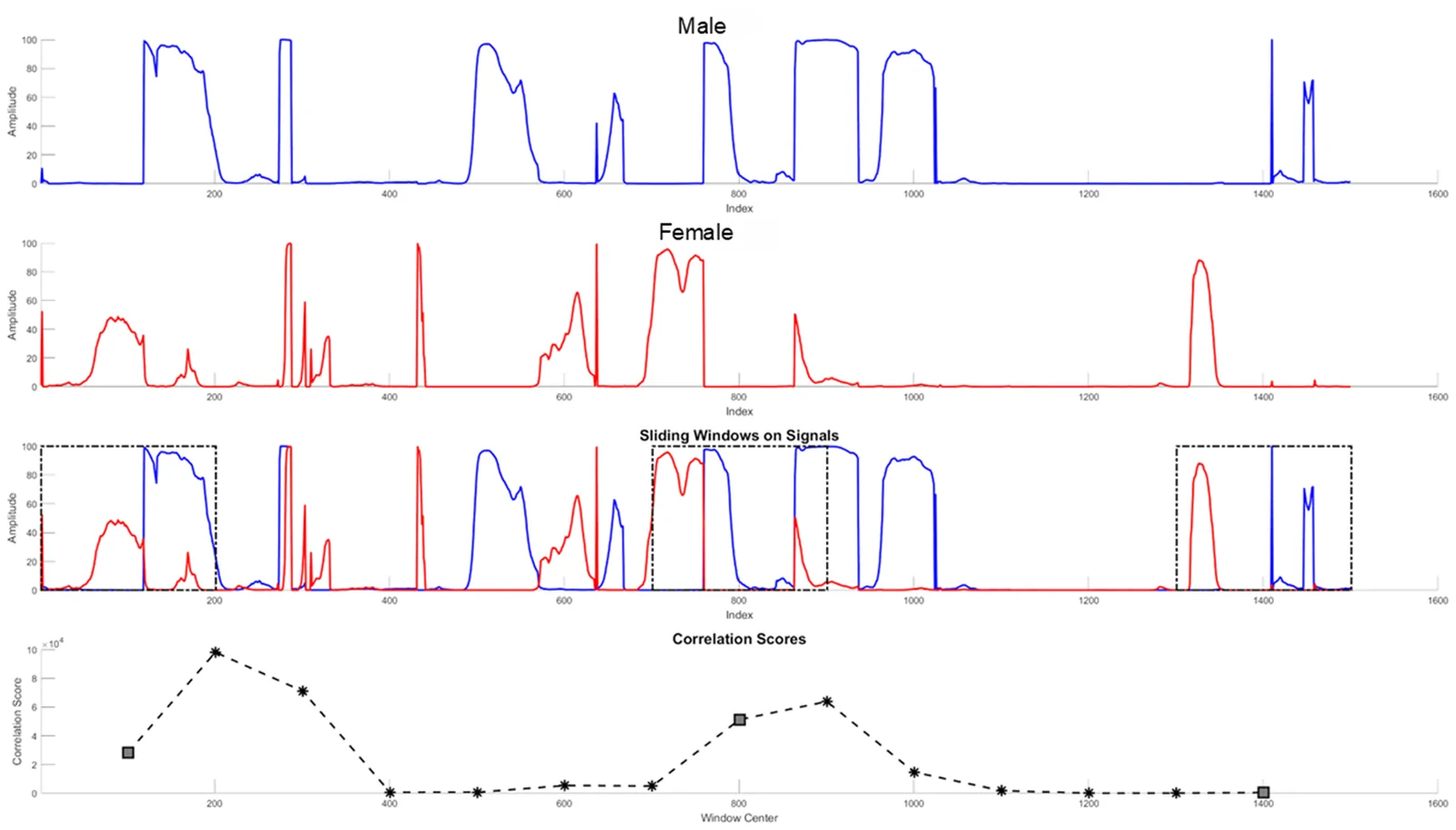
Smile signals of the husband and wife with the corresponding synchrony output (*w* = 200, *st* = 100). From top to bottom: (1) the husband’s smile time series, (2) the wife’s smile time series, (3) both signals plotted together with the sliding window in three positions, and (4) moment-to-moment synchrony scores for each window. Rectangular markers indicate the positions of the windows in the second panel.

**Fig 5:**
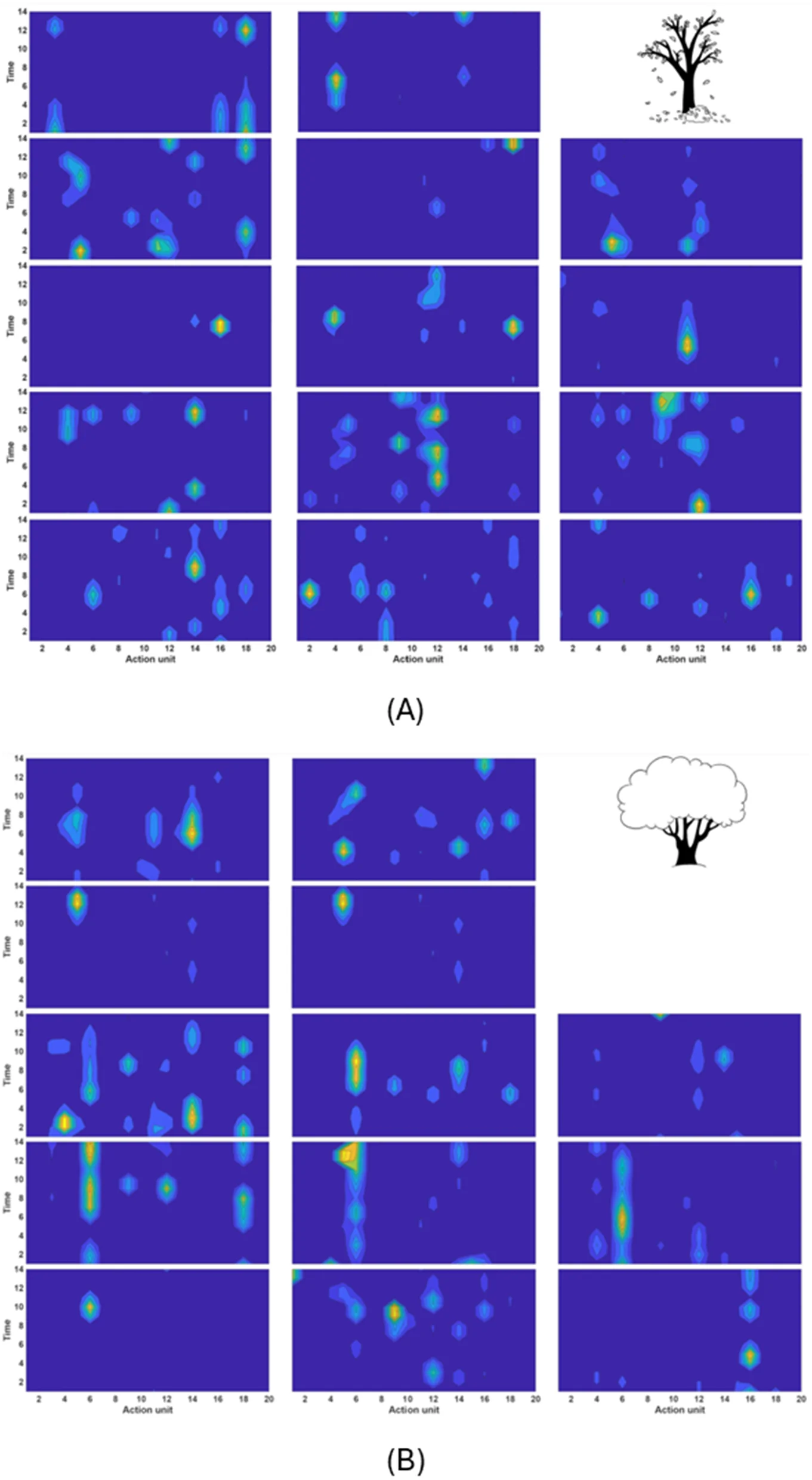
Synchrony profiles for all dyads (*w* = 200, *st* = 100). The horizontal axis represents the index of the action units, and the vertical axis represents the windows. Color intensity indicates synchrony scores, with darker shades corresponding to lower synchrony values. The tree images illustrate the two classes: (A) a tree without leaves represents stressed couples (class 0), while a tree with leaves (B) represents non-stressed couples (class 1).

### 3.5 Performance evaluation via cluster analysis

To evaluate our proposed method, we computed pairwise distance measures based on each dyad’s *S*-matrix and applied a clustering algorithm to reveal separation within the data. We then repeated the clustering pipeline using iMotions-based features proposed in a previous study [36] that does not take into account moment-to-moment variations in facial synchrony. Finally, we compare the performance of the two approaches to demonstrate our contribution.

#### 3.5.1 Pairwise distance measurement.

We concatenated all column (action units) of *S* -matrix into a single vector *v*, refering to this step as flattened *S* -matrix. We then calculated pairwise distances between these vectors using the Euclidean distance, as defined in Eq 3. The pairwise distance computation provides a principled measure of similarity (or dissimilarity) between dyads by capturing differences across the entire synchrony profile, rather than relying on summary statistics or individual action units. In Eq. 3, ${𝐯}_{m}$ and ${𝐯}_{n}$ denote the *flattened S-matrices* corresponding to dyads *m* and *n*, respectively. Each vector is constructed by concatenating all columns of the S-matrix representing action units across all sliding windows into a single vector of length *p*, where *p* is the total number of elements in the S-matrix. The terms $v_{mk}$ and $v_{nk}$ represent the *k*-th elements of the flattened synchrony profiles for dyads *m* and *n*, respectively.


$$\begin{array}{c} d({𝐯}_{m},{𝐯}_{n})=\sqrt{\sum_{k=1}^{p}{\left(v_{mk}-v_{nk}\right)}^{2}} \end{array}$$ (3)

We subsequently applied MDS to project the resulting distance matrix into a two-dimensional space for visualization. MDS preserves the relative distances between points in the low-dimensional space with respect to the original distances in the high-dimensional space. This step facilitates exploration of the latent structure of dyadic synchrony by revealing potential groupings or separations among dyads. MDS assists in identifying patterns and grouping observations, allowing either visual inspection when dealing with a small sample size (like this one) or the application of a clustering algorithm for further analysis.

#### 3.5.2 K-means clustering.

The points distributed in the 2-dimensional space, representing each dyad, were then clustered to detect meaningful underlying differences. Clustering is a machine learning technique that groups similar data points based on their properties, aiming to divide a dataset into distinct groups where items within each group are more similar to one another than to those in other groups. One commonly used approach is K-means clustering, which iteratively assigns data points ($x_{i}$) to *K* clusters based on their proximity to cluster centroids ($\mu_{k}$). The process continues until the cluster assignments stabilize, aiming to minimize the within-cluster sum of squares, as defined in Eq. 4.


$$\begin{array}{c} J=\sum_{k=1}^{K}\sum_{x_{i}\in C_{k}}\|x_{i}-\mu_{k}{\|}^{2} \end{array}$$ (4)

#### 3.5.3 Evaluation metric.

We evaluated clustering performance through both visual inspection and quantitative analysis using internal and external index measures [37]. We used the silhouette score, *s*(*i*), to assess cluster cohesion and separation. For each data point *i*, we calculated the silhouette score using Eq. 5, where *a*(*i*) represents the average distance between point *i* and other points in the same cluster, and *b*(*i*) denotes the minimum average distance between point *i* and the points in the nearest neighboring cluster.


$$\begin{array}{c} s(i)=\frac{b(i)-a(i)}{\max(a(i),b(i))} \end{array}$$ (5)

The silhouette score ranges from −1 to 1, with values closer to 1 indicating well-separated clusters. For external validation, we assessed clustering performance using a confusion matrix, comparing the total number of true positives and true negatives to the total number of data points.

#### 3.5.4 Validation against a baseline approach.

As a baseline, we constructed statistical features from both partners’ iMotions data without using a sliding window. Specifically, we computed all the time-domain features from a previous work [36], including mean, standard deviation, maximum, minimum, kurtosis, skewness, and sum values, resulting in 7 features per action unit per subject. The resulting matrix retains a structural resemblance to the *S* -matrix but does not capture moment-to-moment variations, as the features were extracted from the entire signal without using a sliding window or any moment-based measurements. The extracted features from all action units were concatenated and standardized using z-score normalization, producing a 280-dimensional feature vector (20 action units × 14 features). We compared the performance of the *S* -matrix to this baseline method for detecting relationship quality using the same dataset [28].

## 4 Dyadic QMI threshold selection

Relationship quality was assessed at the dyadic level by summing the QMI scores of both partners. A threshold of 80 was used to categorize couples into “Satisfied” and “Distressed/At-Risk” groups. This threshold emerged from a combination of data-driven inspection and theoretical considerations at the dyadic level, and was used to interpret, rather than define, the structure observed in the data.

First, MDS of the pairwise distances derived from the S-matrices revealed a natural separation into two groups (Fig 7B). Dyads in one region exhibited a clear concentration of lower summed QMI scores, whereas those in the opposing region were predominantly associated with higher scores. Although some higher-QMI dyads appear within the lower-QMI region, the overall spatial organization is not random, as evidenced by the pronounced concentration of lower values within that region. This pattern suggests the presence of two qualitatively distinct groupings. This clustering structure was identified independently of QMI scores, which were subsequently overlaid to interpret the emergent groups rather than to determine them. Second, examination of the empirical distribution of QMI scores supported this separation: the median summed QMI score in the analyzed subsample was 79, and 83 in the full sample of 150 couples, indicating that a threshold near 80 aligns with the central tendency of both datasets, without enforcing an exact or equal split.

Importantly, this threshold is sample-specific and not intended to represent a universal or clinical cutoff. While individual-level **clinical** thresholds for the QMI exist (e.g., ≤29 [38]), marital satisfaction data in **community** samples typically exhibit a negative skew, as highly distressed dyads are less likely to remain in such samples [39]. Combined with the tendency for favorability bias in self-report measures, applying strict clinical cutoffs would result in a highly imbalanced distribution and reduced sensitivity to meaningful variation. Instead, our dyad-level partitioning was designed to enhance the sensitivity of the clustering analysis by distinguishing highly satisfied couples from those reporting comparatively lower levels of adjustment. This approach treats the dyad as an emergent interpersonal system, allowing for the possibility that a couple may function as distressed overall even if one partner’s individual score remains above a traditional clinical threshold. Accordingly, the threshold should be understood as context-dependent and tied to the relational structure observed in this sample, rather than as a transferable benchmark.

## 5 Results and discussion

Conceptually, facial synchrony serves as an observable indicator of coordination between interaction partners, capturing how the dyad adjusts as a coupled system during interaction. From a complex-systems perspective, relationship quality is not expressed through isolated behaviors but through the stability and structure of these interaction dynamics over time. Moment-to-moment synchrony captures local coordination and adaptability that are obscured by aggregate measures, allowing distinctions between dyads whose interactions are consistently aligned versus those characterized by intermittent or unstable coupling. By clustering synchrony profiles in an unsupervised manner, the proposed framework reveals emergent interaction patterns that correspond to relationship quality without imposing predefined relational labels or assumptions.

We examined multiple window sizes while keeping the step size fixed at 4 seconds. The classification performance remained consistent across all configurations, with identical confusion matrices obtained for each window size (Fig 9). To further evaluate clustering quality, we considered both the Silhouette score and the centroid distance as complementary measures of separation (Table 2). The results indicate that the 1000-sample window (40 seconds) provides the best overall performance, achieving both the highest Silhouette score (0.80) and the largest centroid distance ($4.0\times 10^{6}$). Smaller windows, such as 200 samples (8 seconds), show weaker separation (Silhouette = 0.70; centroid distance = $1.1\times 10^{6}$). Intermediate window sizes demonstrate gradual improvement. The 1500-sample (full-length) window yields a slightly lower Silhouette score and a reduced centroid distance, indicating that it does not provide the same level of cluster separation as the 1000-sample case. These results, as summarized in Table 2, suggest that the 1000-sample window offers the most balanced and robust clustering structure. An important limitation is the short duration of the analyzed sessions (~60 seconds), where larger windows (e.g., 40 seconds) cover a substantial portion of the signal (66.7%), potentially reducing temporal resolution.

**Table 2:** Sensitivity analysis of the impact of window size on the Silhouette score using a fixed step of 4s. A window size of 1500 corresponds to the full-signal representation with no overlap. The centroid distance values are rounded to one decimal place.

window size (samples)	window size (s)	Silhouette score	Centroid distance (10^6^)
200	8	0.70	1.1
400	16	0.76	2.1
600	24	0.79	2.9
800	32	0.79	3.9
1000	40	0.80	4.0
1500	60	0.77	2.0

Fig 6 illustrates the distribution of samples in the MDS space with QMI scores overlaid across different windowing configurations. In the figure, key samples that highlight behavior across configurations are circled. The dyad with QMI = 78 consistently appears as a clear outlier across all settings. In contrast, the dyad with QMI = 54 exhibits variability, but aligns more closely with neighboring low-QMI samples under the 1000-sample window, reinforcing the advantage of this configuration. Similarly, the dyad with QMI = 84 remains consistently embedded within the high-QMI cluster, with improved cohesion under the 1000-sample setting.

**Fig 6:**
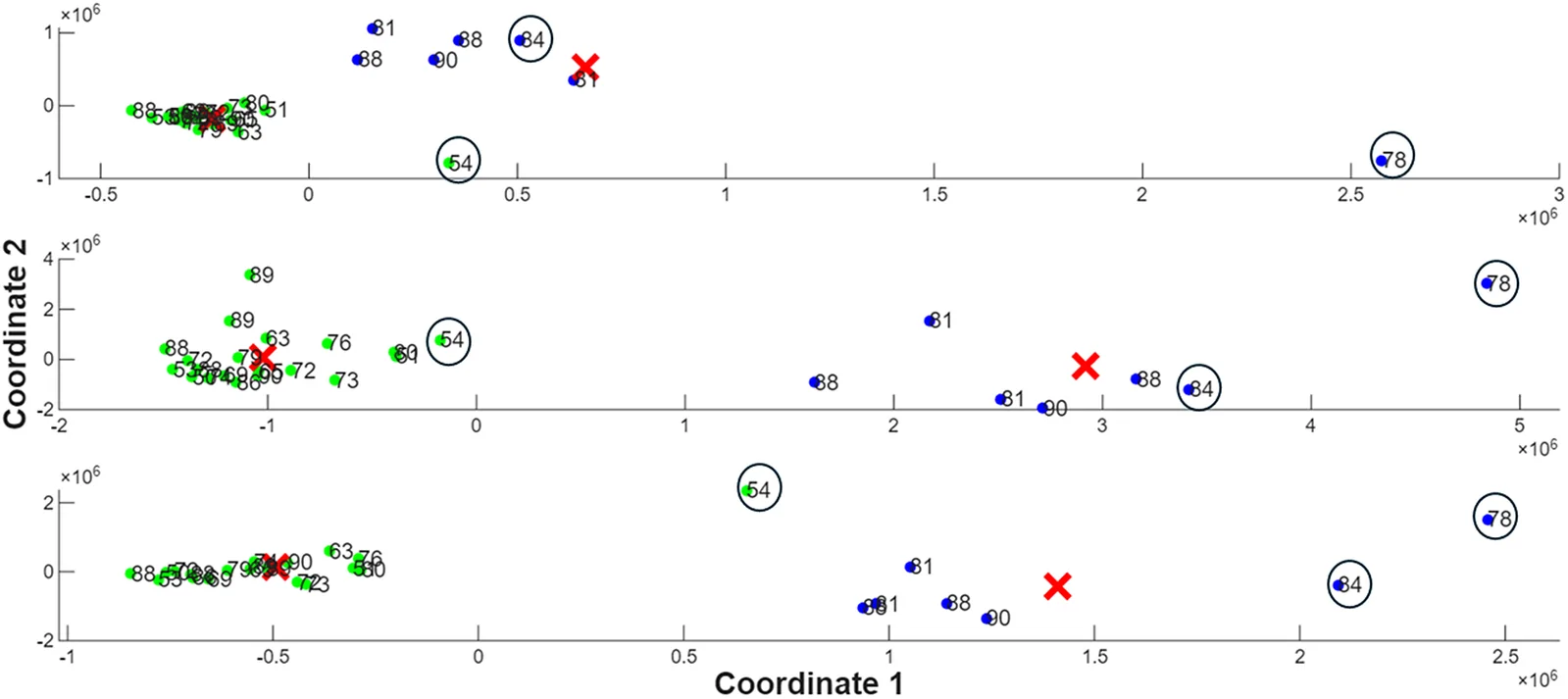
MDS scatter plots of dyadic representations under different windowing configurations. From top to bottom: (1) window size = 200 samples, (2) window size = 1000 samples, and (3) full-window (no segmentation). Points are colored according to k-means cluster assignments, while the numeric labels denote each dyad’s total QMI score. Red crosses indicate cluster centroids, and circled points highlight selected dyads for reference. The figure illustrates how window size influences the clustering structure and spatial distribution of dyads relative to their QMI scores.

Statistical feature extraction was used as a baseline due to its widespread adoption in the literature. From a machine learning perspective, this corresponds to constructing feature representations based on summary statistics. These features were compared with those derived from the synchrony-based approach. Fig 7 shows the scatter plot of labels assigned by the K-means algorithm, comparing the baseline and *S* -matrix (*w* = 1000, *st* = 100) approaches. The *S* -matrix exhibits superior class separation, with a silhouette score of 0.8, compared to the baseline’s score of 0.46, as shown in Fig 8. The group labeled as “stressed” (with low total QMI scores) forms a compact cluster, while the “non-stressed” group (with high total QMI scores) is distributed across two regions: one forming a distinct cluster (red dots) and the other overlapping with the stressed points. This suggests that non-stressed individuals display two distinct behaviors, possibly reflecting dyads heterogeneity or latent factors. To assess whether the two-dimensional MDS representation obscured potential structure, we extended the analysis to three dimensions. The resulting configuration showed a pattern largely consistent with the two-dimensional embedding. Notably, the third dimension provides slightly improved visual separation for certain high-QMI dyads, which appear distinct from the lower-QMI region. However, for larger window sizes, this pattern is already evident in 2D, and the same overall separation pattern between two groups is preserved across window sizes. Clustering was performed using K-means as a simple proof-of-concept. Metrics such as silhouette score and centroid distance depend on the clustering assignments and may not fully capture localized structures. Given the consistent visual pattern across 2D and 3D, the two-dimensional representation was retained for clarity and interpretability.

**Fig 7:**
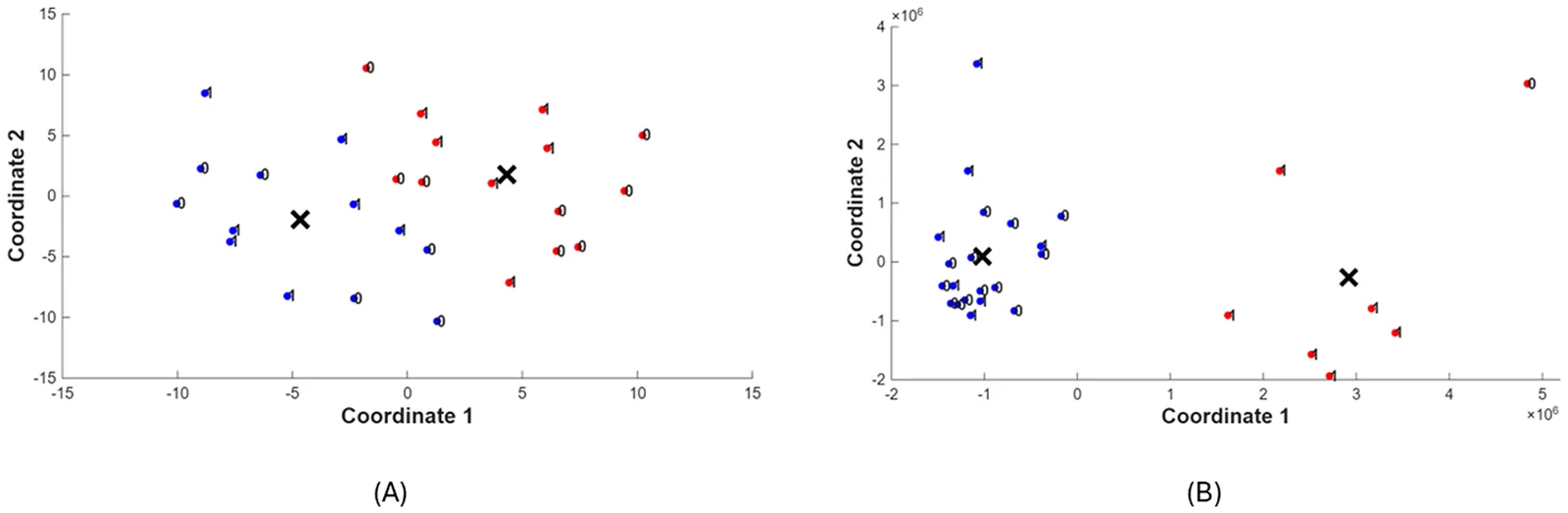
Scatter plot of observations in two dimensions, where the number on each point denotes its true class, and the color indicates the cluster assigned by K-means algorithm. (A) using the baseline approach, (B) using the windowed cross correlation (*w* = 1000, *st* = 100).

**Fig 8:**
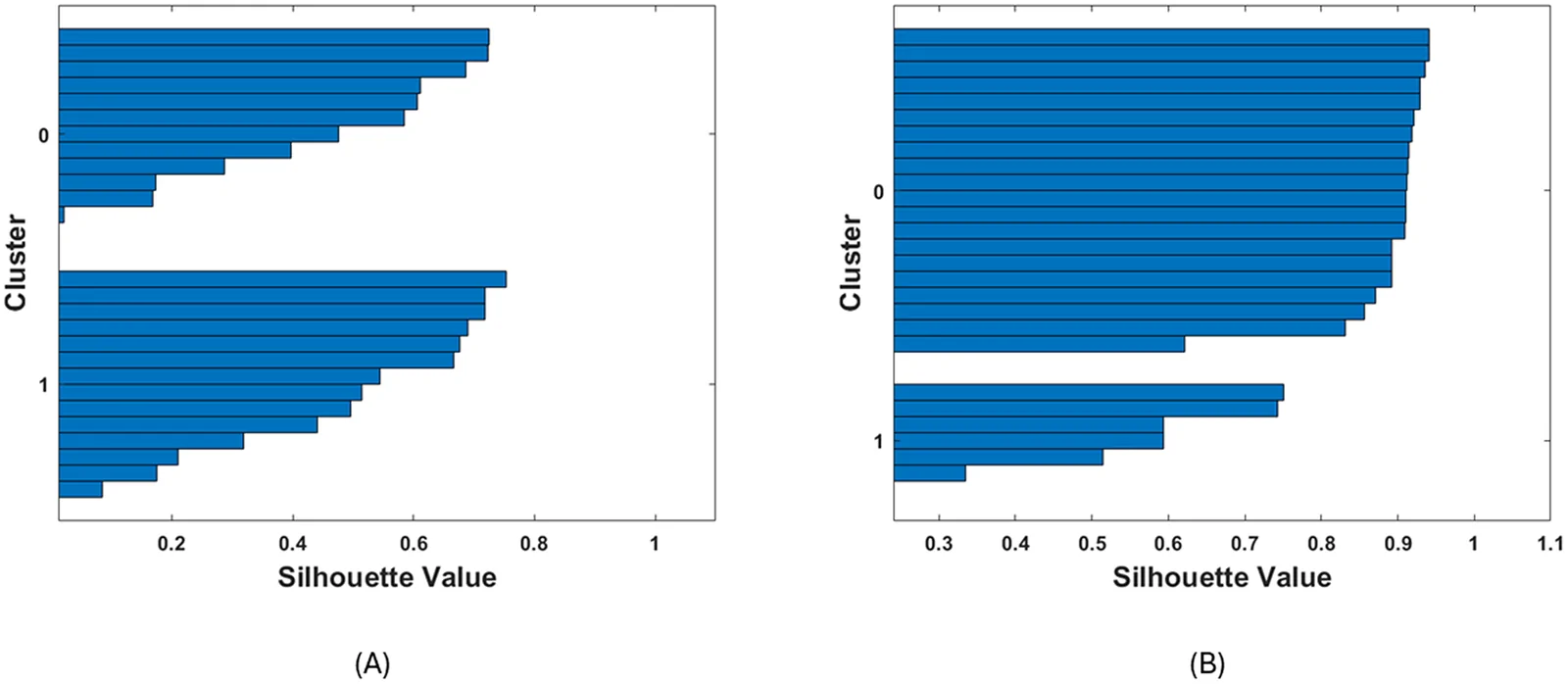
The Silhouette plot showing the silhouette score for each observation. (A) using the baseline approach, (B) using the windowed cross correlation (*w* = 1000, *st* = 100).

While the no-window synchrony method does not outperform the windowed approach, it still demonstrates advantages over the statistical baseline, particularly in the emergence of structured patterns in the embedding space. Notably, although the false positive rate is similar for both the statistical and no-window synchrony approaches—as shown in the confusion matrix (Fig 9)—the scatter plots reveal clearer and more coherent grouping when using synchrony. In contrast, the statistical features produce more diffuse and less separable clusters. A key distinction between the two approaches lies in feature dimensionality. For the statistical method, extracting *n* summary measures across 20 action units yields a feature space of size 20×n. The use of windowing further increases dimensionality, potentially leading to high-dimensional representations relative to the available data. In contrast, the synchrony-based approach, implemented via zero-lag cross-correlation, produces a more compact representation by generating a single value per action unit per window (20×1). This enables a direct comparison between a high-dimensional statistical baseline and a lower-dimensional synchrony-based feature set. Overall, this comparison highlights that while statistical features serve as a conventional baseline, the synchrony-based representation captures richer relational structure. The no-window synchrony case provides an intermediate baseline, and the full benefit is achieved when synchrony is combined with an appropriate window size, particularly the 1000-sample configuration.

**Fig 9:**
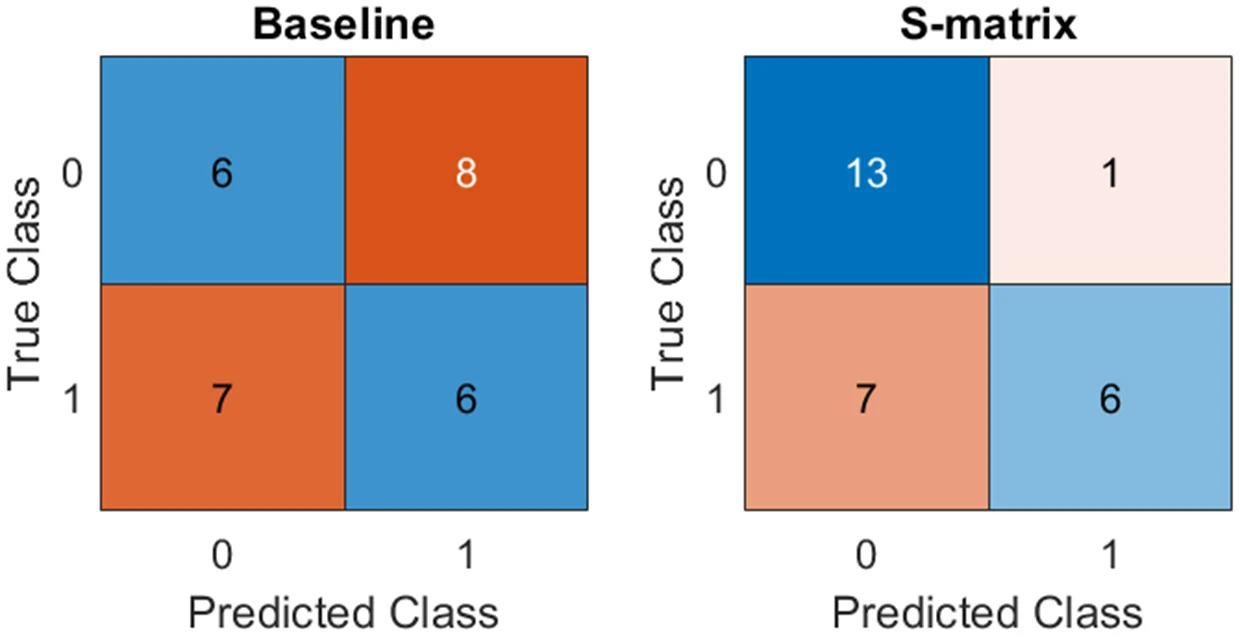
Confusion matrices for clustering performance using the baseline approach and synchrony profiles. A group label of 0 represents the stressed group, whereas a label of 1 represents the non-stressed group.

Window selection requires a balance between capturing long-term trends and short-term fluctuations. A 40-second window with a 4-second shift results in fewer segments, leading to smoother features but potentially introducing redundancy due to higher overlap. In contrast, an 8-second window generates more segments, increasing sensitivity to short-term variations, but may sacrifice context. A summary of these comparisons is provided in Table 3. To enhance the visualization of synchrony profiles within and between dyads, we normalized the profiles to the [0,1] range for each window, which aids in interpreting topographic contours of the *S* -matrix. Fig 10 displays the synchrony profile for Dyad 14, a non-stressed dyad, using both 8-second and 40-second windows. As shown, the shorter window captures more granular structure with time, while the longer window produces a smoother profile.

**Table 3:** Summary of the findings in this study.

Parameter	*w* = 40s	*w* = 8s
Number of Segments	$\frac{60-40}{4}+1=6$	$\frac{60-8}{4}+1=14$
Overlap Percentage	90%	50%
Temporal Resolution	Low	High
Feature Stability	More stable	More dynamic
Computational Cost	Lower	Higher

**Fig 10:**
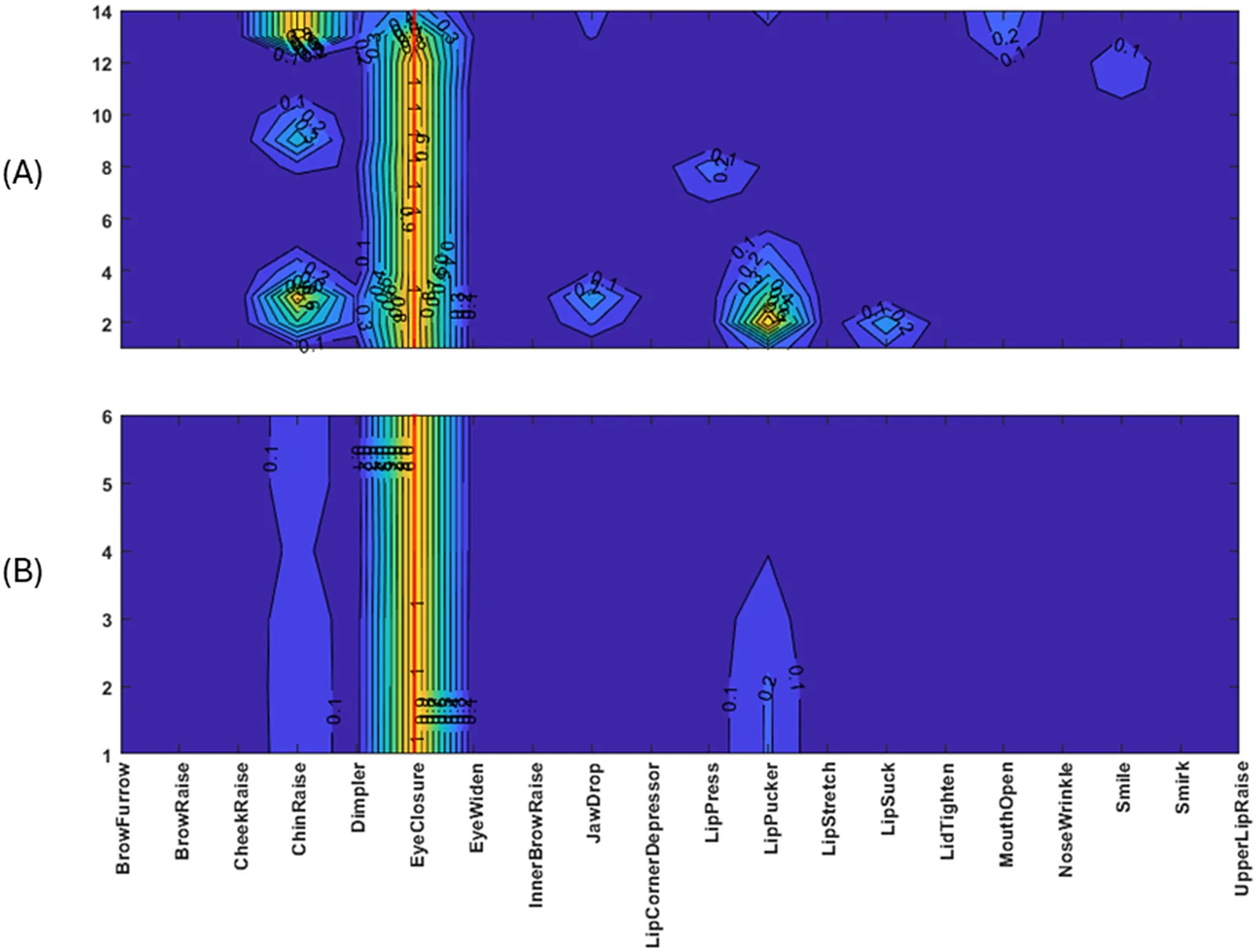
Spatiotemporal representation of the normalized synchrony profile for Dyad 14 (Class 1: non-stressed relationship). The sixth column (eye closure action unit) is marked in red line, reflecting its increased intensity in some Class 1 dyads. The figure compares using two window sizes ((A) *w* = 200, and (B) *w* = 1000), with the shorter window capturing finer temporal details.

The synchrony profiles for the right-side observations in Fig 7(b) showed a higher intensity in the sixth column (highlighted in red in Fig 10), corresponding to eye closure, when compared to other action units. Eye closure (AU43), associated with the relaxation of the levator palpebrae superioris muscle, suggests that this action unit occurs more frequently in both individuals than by chance, indicating potential eye related movement which is a key nonverbal cue. For example, Wohltjen and Wheatley [40] used pupil data to examine mutual eye contact and found a correlation with engagement ratings. Unlike tracking the pupil, monitoring AU43 with conventional cameras eliminates the need for specialized equipment to track the subjects’ pupils [41]. Therefore, utilizing AU43 could be seen as an indicator of communication. In fact, research highlights the communicative value of eye closure, signaling attentiveness, emotions, and responses [42].

The improved performance of the S-matrix approach suggests that the variability and sequencing of synchrony are perhaps more diagnostic of relationship health than its mere presence. Specifically, the clusters identified in this study may reflect relational phenotypes-distinct patterns of non-verbal attunement that distinguish resilient systems from those in distress. Traditional aggregative measures assume that more synchrony is inherently better. However, coregulation theory suggests that healthy dyads are characterized by the ability to dynamically synchronize during moments of shared affect and de-synchronize when that level of attunement is less necessary, patterns which the S-matrix is uniquely equipped to identify. However, whether or not the patterns of synchrony are a precursor to or consequence of marital distress remains an interesting theoretical question requiring longitudinal methodologies.

## 6 Conclusion and limitations

In this study we analyzed moment-to-moment facial synchrony by constructing synchrony profiles using multidimensional zero-lag cross-correlation. Leveraging spatiotemporal representations, we successfully differentiated between distressed and non-distressed couples based on their total QMI scores. The proposed approach outperformed traditional feature fusion methods, achieving a higher silhouette score of 0.8 compared to 0.46. These findings highlight the significance of automated facial expression analysis and its potential applications in psychology and relationship research. By moving beyond static correlations toward a multidimensional synchrony profile, this approach offers a path toward objective diagnostic tools in clinical settings, potentially identifying early signs of relational decay before they manifest in self-report measures, or as pathognomonic of existing distress. In either case, an automated, synchrony-based profile provides a non-invasive, high-resolution and objective marker for relational health. In a clinical context, this approach could help provide greater precision by allowing clinicians to identify moments of affective decoupling during a session; however, this should be viewed as a proof-of-concept rather than evidence of immediate clinical applicability.

A key limitation of this work is the need for a larger dataset and longer valid sessions to best validate the method and determine the optimal window and step sizes. Additionally, the choice of Euclidean distance for calculation may not be ideal in higher-dimensional spaces, and alternative distance metrics should be explored. While eye contact is a significant cue, it should not be analyzed in isolation, as other action units also play a role in communication. Future research should focus on examining the interactions between action units to gain a deeper understanding of facial communication dynamics. As a first step, we focus on same-time coupling to establish a clear and stable baseline for dynamic facial synchrony. Extending this framework to incorporate moment-to-moment lagged and directional dynamics is a natural next step and will be addressed in future work. A further limitation is the use of dichotomized QMI scores, which simplifies relationship quality; future work should examine it as a continuous measure as well.
