# Supplementary material for: Characterizing multidimensional facial synchrony for relationship quality detection
Source: PLoS One. 2026 Oct 1;21(10):e0356071. doi: 10.1371/journal.pone.0356071 (PMC13630251; doi:10.1371/journal.pone.0356071)
Supplement: Supplementary file 1 [file pone.0356071.s002.pdf]

Review of manuscript PONE-D-25-41087

Overall assessment

I thank the authors for addressing the issues brought to their attention by myself and another reviewer. I think that, overall, the authors have been accommodating in addressing the suggestions and concerns, such as making the data and code available in open repositories and providing the visual overview presented in the new Figure 1. This figure will certainly make it easier for readers to digest the paper and will remind them where each step fits into the overall method. It also seems that the provided code successfully replicates the results, which should be a great help for anyone wishing to apply the method in their own work.

I want to thank the authors for providing details on the data and preprocessing that was previously missing.

Since PLOS One does not provide copyediting the authors should have the manuscript copyedited before submitting a revised version.

Major comments

Overall, I only have two major comments, that I think it would be pertinent to address in a revised manuscript. These comments relate to (i) the selection of the QMI threshold and (ii) the baseline comparison. Both issues were also included in my original review, but with the revised manuscript and the authors' replies, I hope to be able to get my point across. Each of these issues are described separately in the following.

QMI Threshold

In my original review I hypothesized that the QMI threshold of 80 was selected in order to dichotomize the quality of marriage into two groups of roughly equal size. While I think the revised text and the authors' response to my initial review amount to a confirmation of this hypothesis, the authors stop short of stating this explicitly in the manuscript. Since I am not completely certain, I will entertain the consequences of both outcomes below.

If my hypothesis is true, then I think it should be stated explicitly in the manuscript. However, this leads to the logical next question: would a threshold of 80 also result in an equal split if the whole sample of 150 couples? If yes, then all is fine, but if not, then what are the implications? It would be nice if the authors could check this, and add the results to the manuscript.

If, on the other hand, my hypothesis is false and the QMI threshold was selected based on other criteria, then the rationale for selecting the QMI threshold of 80 is still unclear. I understand the argument against the commonly applied clinical thresholds, but did not understand HOW the chosen threshold was obtained, only that it "was informed by both the distributional properties of our

community sample and our theoretical focus on the dyad as the primary unit of analysis." But is is unclear to me exactly what is meant by this statement.

I am aware that this may seem like a small point to belabor, but I fear that providing a threshold without providing the process for arriving at it risks that others will then later copy this threshold. But if we (and more importantly they) do not know whether this threshold is expected to be universal or transferable to other similar samples, then we may end up simply replicating the number — because that is all that we are given. Instead, I hope that the authors can share their method and their thinking with us, so that we may use their method, and not just their number.

I see some traces of the process in the paper (Overleaf version, p. 8 l. 200-204): "Given this skew and the common tendency for favorability bias in self-report measures, a strictly clinical cutoff would have resulted in a highly unbalanced distribution. Instead, our dyad-level partitioning was designed to facilitate the sensitivity of the clustering algorithm by distinguishing highly satisfied couples from those reporting lower levels of adjustment."

Baseline comparison

In the ultimate paragraph of the "Results and discussion" section the authors write: "The superior performance of the S-matrix approach . . . " However, bear in mind that this superior performance is relative to the simplest possible baseline — something I also addressed in my initial review.

In their reply the authors write: "Given the limited continuous duration available in the current dataset, such comparisons could not be explored exhaustively and are therefore identified as important directions for future work." I must admit that I do not understand why the limited continuous duration is a barrier to comparing to aggregate measures. This is exactly what is done in the current baseline approach, just using traditional statistical measures. What I meant was that it would be more interesting to compare to an existing aggregate measure of synchrony.

In fact, the authors could simply set the window size to 60s and the step size to 0 which would give a non-windowed version of the method. Then it would be simple to compare this to the current 40s sliding window approach to see how much they differ.

In lieu of a more appropriate baseline, some readers might be justified in thinking that the baseline was chosen in order to make the comparison favor the authors' proposed method.

Minor comments

I have some minor comments that can easily be implemented in a revised manuscript, which I offer for the authors' consideration.

Add explanation for exclusion earlier

In section 3.1 you write:"The initial data set comprised ≈ 150 engaged heterosexual couples; however, 27 couples were selected for analysis."

I think this sentence needs to be elaborated a bit, or modified to let the reader know that an explanation is coming later in the manuscript for why only 27 of the 150 couples were included. Also, it would make sense to include the word "only" before "27 couples".

Figure 2

Figure 2 clearly shows why 60 seconds is a reasonable choice to compare the available dyads. Since the dyad ID's on the horizontal axis in Figure 2 has no intrinsic ordering, I would suggest sorting the dyad ID's by the length of the available synchronized data, to make the plot easier to interpret.

K-means clustering results

In the discussion of Figure 7 the authors write: "the "non-stressed" group (with high total QMI scores) is distributed across two regions: one forming a distinct cluster (red dots) and the other overlapping with the stressed points. This suggests that non-stressed individuals display two distinct behaviors, possibly reflecting dyads heterogeneity or latent factors."

I can think of at least two alternative explanations:

1. Using MDS to reduce the data to two dimensions does not properly represent the data. It may be that the structure would separate better if a third dimension was added, but this is pure speculation. However, unlike heterogeneity and latent factors, this is something that can easily be investigated.
2. The QMI threshold could be adjusted to a more optimal value. It would be interesting to see a version of Figure 6 where the value of QMI was added to each data point. Are the mis-categorized couples' QMI close to the threshold? If so, then the dichotomization of marriage quality may be the reason. In fact, I made a slight modification of the authors' code to plot `labels_num` instead of `idx` (see attached plot) and while the point labelled "0" in the upper-right corner is very close to the threshold (it has QMI = 78) this does not seem to be the case for the points labelled "1" in the left-hand cluster. In other words, this explanation seems to be ruled out.

Report window sizes

The authors briefly discuss the effect of selecting different window sizes, and tabulate the silhouette scores for different window sizes. However, this does not directly inform as to whether the results change or not. The authors write

that “The performance was consistent across the various window sizes, as shown in the confusion matrix (Figure 8)... ” I don’t understand how the confusion matrix can show this, since it is for a single window size.

And this leads me to the second point about window sizes: In most cases the authors omit reporting the window size used for results and figures. Unless I missed it, the authors do not state what window size was used in cases where it is not explicitly stated. But from the MATLAB code, it seems that the analyses were performed with a window size of 1000 samples or 40 s. Please state this somewhere in the manuscript and also in all figure captions. It is particularly important in Figure 9, where two different window sizes are compared, but they are not stated in the caption.

Practical implications and limitations

If I were to summarize the results of the paper, very briefly, I would say that the authors are clearly on to something: Their MDS-reduced flattened S-matrix distance measure can indeed be used to form distinct clusters using k-means clustering. While the results are not perfect, they seem to capture at least some of the variation in the self-reported QMI measures.

The authors state that “In a clinical setting, this could allow for greater precision, where clinicians can pinpoint the exact moments of affective decoupling during a session.” I have not seen any convincing evidence in support of this statement. I am not convinced that there is a good case for clinical applications — at least at this stage, and again I return to my initial suggestion of approaching this in terms of continuous measures instead dichotomized ones. This is something the authors do not seem to acknowledge as a limitation, but relying on binary classification says nothing about the degree of marriage quality. A case in point is the confusion matrix, which indicates that the method is no better than the baseline for non-stressed couples. So the false positive rate is quite high, provided, of course, that we accept the self-reported QMI as the ground truth. It may not be.

Other minor issues

- Line after Eq. 6: A hyphen is used instead of a minus sign: “The silhouette score ranges from -1 to 1, ... ”
- Figure 5:
  - Caption for Figure 5 refers to x-axis and y-axis, but neither x nor y are variables in the plot. Use abscissa and ordinate or horizontal and vertical axes instead.
  - The caption refers to (A) and (B) but these are not shown in the figure. Instead there are images of a tree without leaves and with leaves, respectively.

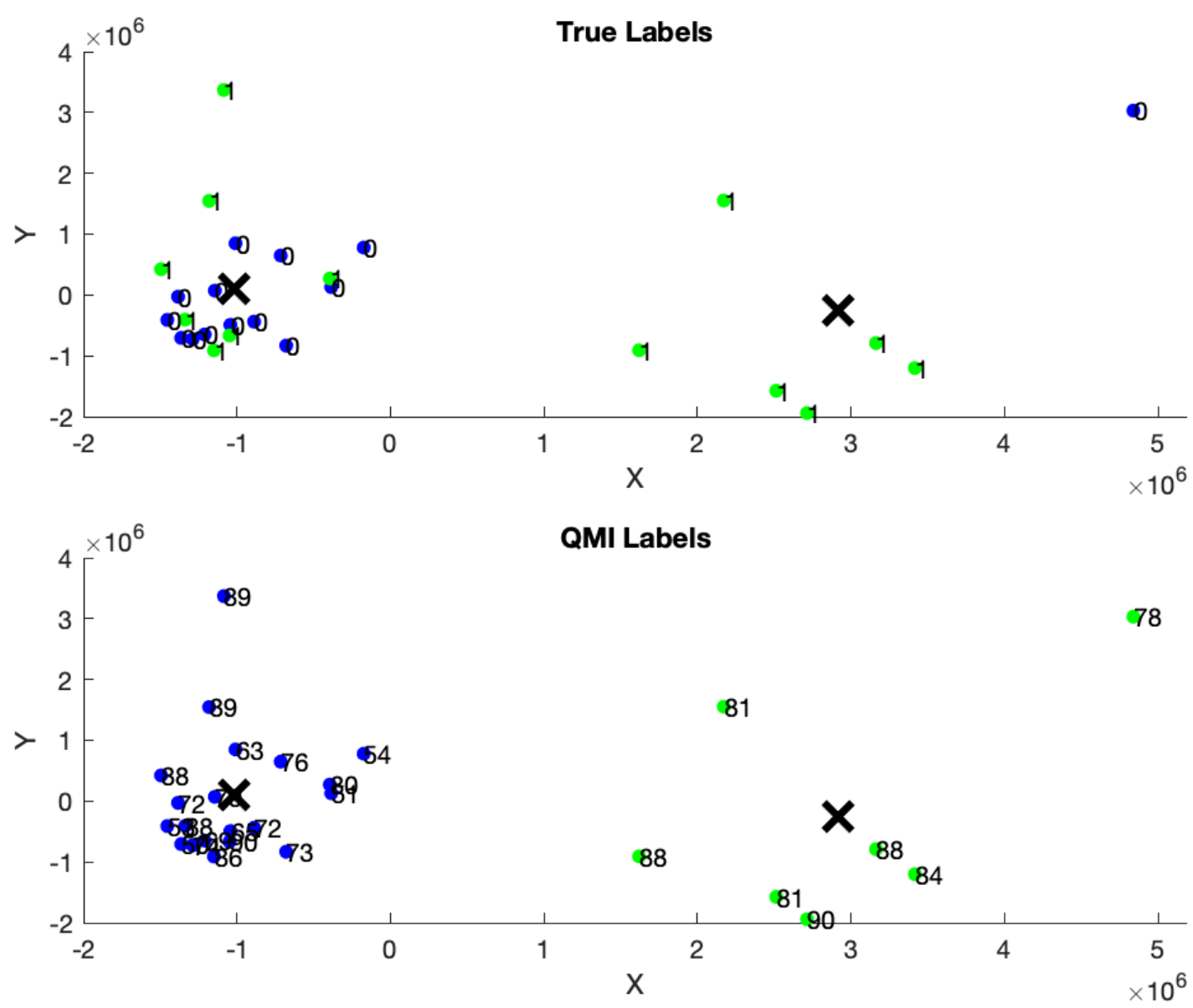
True Labels
QMI Labels
X
Y
×10^6
