# Supplementary material for: Characterizing multidimensional facial synchrony for relationship quality detection
Source: PLoS One. 2026 Oct 1;21(10):e0356071. doi: 10.1371/journal.pone.0356071 (PMC13630251; doi:10.1371/journal.pone.0356071)
Supplement: Supplementary file 2 [file pone.0356071.s001.docx]

We thank the Editor and the two Reviewers for their careful reading of our manuscript and for their constructive and insightful comments. We have carefully considered the Editor’s comments as well as those of both Reviewers, and we have revised the manuscript accordingly. Below, we reproduce each reviewer’s comment followed by our detailed response. For ease of tracking, all responses are shown in red.

In response to the reviewers’ request for improved reproducibility, we have attached the full analysis code as supplementary material. All processed datasets are provided in MATLAB .mat files, and the complete preprocessing and analysis pipeline is implemented in MATLAB .m files. These scripts serve as replication code and allow third parties to reproduce the results reported in the manuscript. The source data files in their original format (.csv) are archived and publicly available on Zenodo to ensure long-term accessibility and transparency. Both the source data and the analysis code are provided via two separate url links.

# Review #1

1. Actual numerical results are reported in the Introduction (line 32). Numerical findings should not appear in this section.

Thank you for this comment. We have revised the manuscript accordingly and removed numerical findings from the Introduction.

2. The claim that this is “the first study to characterize moment-to-moment synchrony in facial expressions for relationship quality detection using such a comprehensive and unsupervised approach” (lines 33–35) is overstated. Prior studies have already examined naturally occurring facial synchrony as an indicator of relationship quality in various interpersonal contexts, including Machluf-Ruttner et al. (2024), Rohr et al. (2022), Yokotani et al. (2020), and Cheong et al. (2023). The claim of novelty should be moderated accordingly.

We thank the reviewer for pointing out that the original novelty claim was overstated and for referencing relevant prior work on facial synchrony in interpersonal and relational contexts. We agree with this assessment and have revised the manuscript accordingly. The novelty statement has been rewritten to emphasize that the contribution of this work lies in introducing a fully automated, unsupervised machine-learning framework for marital relationship quality detection based on dynamic, moment-to-moment facial synchrony, rather than in being the first to study facial synchrony per se.

3. The term “Action Unit (AU)” is abbreviated at the start of the introduction (line 25) and used throughout the manuscript. When used as part of the text, it should appear in full as “action unit,” whereas in technical contexts referring to specific units (e.g., AU1, AU2), the abbreviation is more appropriate.

Thank you for this helpful clarification. We have made edits accordingly throughout the manuscript.

4. Typo on line 213: extra space before the period in “...expressions over time .”.

Thank you for the comment. We have fixed the typo.

5. Figures require revision:

- Figure 1 may be unnecessary given the clarity of the textual explanation.

Thank you for this comment. We agree with this assessment and we have removed Figure 1.

- The title of Figure 2 uses an internal label that is inappropriate for publication.

Thank you for this comment. We have removed the internal label and made everything clear in the caption.

- Image quality across figures is poor and should be improved through direct export rather than screenshots.

Thank you for this comment. We have updated all figures to comply with the journal’s figure specifications.

- Figures 2, 3, and 8 show only a single dyad; if possible, data from the full sample should be represented.

We thank the reviewer for this valuable suggestion. Figures 2, 3, and 8 (as numbered in the previous version) present results from a single representative dyad and are included for illustrative purposes, enabling a clear and interpretable demonstration of the proposed methodology. To ensure transparency and reproducibility, data from all dyads are publicly available for interested readers. In addition, Figure 5 (formerly Figure 4) presents synchrony profiles for the full sample, thereby capturing the overall patterns observed across all dyads and demonstrating the consistency of the proposed approach at the group level.

- Axis and legend labels should be consistently capitalized.

Thank you for this comment. We have capitalized all axis and legend labels.

- Some figures lack sufficient explanatory detail in their captions (e.g., Figure 7).

Thank you for this comment. We have provided more detail in the caption.

6. The manuscript places heavy emphasis on methodological considerations, such as windowing and clustering, while providing limited discussion of the conceptual implications. The theoretical meaning of linking facial synchrony to relationship quality in this way could be developed more clearly and prominently.

We thank the reviewer for this insightful critique. We agree that while the technical advancements of the S-matrix approach are significant, its value is ultimately realized through its ability to illuminate the psychological processes underlying marital health. In the revised manuscript, we have significantly expanded the theoretical framework in the Introduction and Discussion sections. Specifically, we now frame facial synchrony not merely as a correlate of satisfaction, but as a dynamic marker of interpersonal attunement and communication flexibility. We have also added practical implications. More specifically, we now discuss the potential for this method to serve as an objective digital biomarker in clinical settings. These additions help bridge the gap between our systems-science methodology and the interpersonal-science theory it supports.

# Reviewer #2

**Replicability**

I commend the authors for making the full dataset available. I downloaded the set of files provided in the hope that I could also replicate the results in the article by following the description in the methods section. However, I quickly realized that (as far as I can tell) there was no information about how the female and male participants were matched in couples. A guess is that Dyad = Respondent - Gender, but this leaves about a dozen unmatched participants, so I am not certain.

The remedy for this is simple: The authors should provide one or more replication scripts with the code for the analysis performed in the paper. This can be in any software the authors have used such as R, Python, Julia, Octave/MATLAB, etc. Please also ensure that both data and replication scripts are available in a public repository such as Zenodo, Open Science Framework, figshare or similar.

We thank the reviewer for this comment and apologize for the lack of clarity regarding dyad–partner matching. We have now provided a self-report table that explicitly lists the dyad ID, and the corresponding individual QMI and other self-report scores, making the couple matching unambiguous.

We have also included replication scripts with all required data as supplementary materials for the review process. The Zenodo repository currently hosts the original dataset for general reuse; we will release an updated version including the dyad-mapping file and replication code, or archive these materials with the final supplementary files upon publication.

We appreciate the reviewer’s suggestion, which helped strengthen the reproducibility of this work.

**Dichotomizing the variables**

The QMI score is dichtomized by what appears to be a median split (although this is not explicitly stated). The description on p.6, l.142-146 indicates that the threshold of 80 was selected to obtain an even split. If this was the reason, please write this explicitly, or provide an alternative reason.

We thank the reviewer for identifying the need for a more explicit rationale regarding our scoring threshold. The choice of a dyad-level threshold of 80 (equivalent to an average individual score of 40) was informed by both the distributional properties of our community sample and our theoretical focus on the dyad as the primary unit of analysis. While clinical benchmarks – such as the score of greater than or equal to 29 suggested by Woods et al. (2013) – are useful for identifying severe individual distress, community samples of married couples are characteristically skewed toward higher satisfaction due to marital selection effects—where highly distressed couples exit the population via separation or divorce.

In our sample, a strictly clinical cutoff would have resulted in an extremely unbalanced partition, failing to capture the "at-risk" or "moderately dissatisfied" couples who still exhibit meaningful variance in facial synchrony. By using a threshold of 80, we partitioned the sample into "Satisfied" versus "Distressed/At-Risk" dyads. This approach provides the clustering algorithm with enough signal to distinguish between highly adjusted systems and those experiencing relational strain, which is more appropriate for a community-based detection model. We have updated the manuscript to state this rationale explicitly.

More fundamentally, dichotomizing the dependent variable like this discards data and variation within the data that is then no longer available in the analysis. I suspect this was done to simplify the analysis, but it would be interesting to see results of an analysis where this information was retained, e.g. a multiple linear regression analysis with the (un-dichotomized) dyad-level QMI. Similarly the independent variable is dichotomized by requiring two clusters in the k-means clustering step. There are alternatives, such as logistic regression, where the independent variable can be retained, even if the dependent variable is dichotomized. It would be instructive to see results of an analysis where neither the independent nor the dependent variable have been dichotomized. There may be good reasons to do this, but it should not be done as the first step and the reasons should be provided to the reader.

Thank you for this thoughtful comment. We agree that analyses retaining continuous QMI scores and continuous synchrony measures are informative when the primary goal is prediction or parameter estimation. The aim of the present work, however, is structural characterization and similarity analysis of dyadic interaction dynamics, rather than supervised prediction. Accordingly, we focus on pairwise distance measures between continuous synchrony profiles and their low-dimensional representations to examine relational structure among dyads.

The synchrony features are not used to *predict* QMI, nor are they discretized to fit a classifier. Instead, each dyad is represented by a continuous, high-dimensional synchrony profile derived from sliding-window zero-lag correlations, which preserves temporal variability throughout the analysis. Clustering was used as an additional structure-discovery step, rather than as a discretization of the independent variable or a substitute for continuous modeling. Specifically, k-means clustering was applied as an unsupervised, exploratory method to identify dominant dynamical regimes in the space, without imposing thresholds or class labels. The choice of *k* = 2 was aligned with the outcome grouping and further supported by visual inspection, serving to formalize an emergent pattern rather than to force a dichotomy. We have updated the manuscript to state this rationale explicitly.

**Motivation for the method**

In brief, the method takes simultaneous videorecordings of individuals in a couple and use an automated algorithm to extract AU activation for each AU and each frame of the video. This gives a time series of AU activations that can be matched up for the two individuals in each dyad. These are used to construct a so-called S-matrix of zero-lag correlations between AUs (rows) in a specific time window, and time windows (columns) in a sliding window approach where the window of width w, is moved by some step size along the time series. In a next step pairwise Euclidean distances between vectors that are columns of the S-matrix are calculated and subjugated to multidimensional scaling (MDS) to obtain a two-dimensional representation. The data points in this two-dimensional space are then used as input to a k-means clustering algorithm fixed at producing two clusters from the data. The cluster membership of each couple is then compared to the dichotomized quality of marriage index (QMI) categorizing couples as either stressed or non-stressed.

There are several places along the way, when I read the description of the method summarized above, where I think a little motivation would help to understand the method. I constantly kept wondering why the authors made some very specific choices where there were other alternatives I could think of. I do not ask the authors to consider such alternatives in detail, but merely to provide some reasoning for the steps in the analysis. A figure explaining the method in a conceptual way could go a long way to increase the readability of the manuscript, e.g. along the lines of Figure 1 in Shadeydeh et al. (2021).

Thanks for this great suggestion. We have attached a figure shows the main steps of the work as suggested, together with reasoning for the steps. Briefly, the use of cross-correlations between AUs was motivated by our goal of quantifying facial synchrony within dyads. Applying this analysis in a sliding-window framework allowed us to capture the temporal dynamics of synchrony over the course of the interaction, rather than assuming stationarity. The resulting *S*-matrix can thus be interpreted as a time-resolved signature of dyadic interaction patterns.

To assess similarity between dyads, we computed distances between these *S*-matrix profiles, yielding a distance matrix that quantifies how similar or dissimilar different dyads are in terms of their interaction dynamics. Multidimensional scaling (MDS) was then used to obtain a two-dimensional representation of these distances, facilitating visualization and subsequent clustering, as well as comparison with relationship quality measures.

**Some of the choices I would like to see explained are:**

i) What is the purpose of calculating the pairwise distances between the columns of the S-matrix?

Pairwise distances between S-matrices are computed to quantify how similar or dissimilar dyads are in their overall multivariate, time-varying synchrony patterns, enabling visualization and clustering of interaction dynamics without relying on individual features or predefined labels. We have updated the manuscript to address this point.

ii) why do the authors use MDS to perform dimensional reduction when k-means clustering is fully capable of operating on higher-dimensional data.

Thank you for this insightful question. We can apply k-means to S-matrices, but not directly in their raw matrix form unless we handle the matrix as image or extract the pattern from matrix. We need a representation where each S-matrix becomes a vector or a point in a space where distances are meaningful. MDS is used to obtain a low-dimensional, distance-preserving representation of dyadic synchrony patterns that improves interpretability and robustness, while k-means is applied subsequently for clustering within this embedded space. In sum, the goal of MDS is to see the emerging behavior of dyads in low dimension where we were able to recognize it even by visual inspection.

iii) I also wonder what would happen if a different number of clusters had been found. I understand that the authors are interested in having two clusters to be able to compare to the dichotomized QMI score, but at least a consideration of the number of clusters seems appropriate to include.

We thank the reviewer for this comment. The number of clusters was fixed to two a priori, as the study is theoretically grounded in a binary distinction between stressed and non-stressed states reflected in the QMI outcome. Given the small sample size (27 dyads) and the clear visual separation observed in the low-dimensional representation, our goal was not to estimate an unknown number of clusters but to test whether the data structure aligns with this predefined dichotomy. We added statement showing this rationale in the manuscript.

**Description of the method**

Apart from the reasoning behind the method, I would also like to see it described a bit more concisely. In several places I had to stop and think about exactly what was meant by a particular phrase or expression. Here are a few examples:

p.8, l.198: "where X_j and Y_j represents the AU vectors of the two individuals within the window w_i." This makes sense, but it is the first time the reader encounters X_j and Y_j and w_i has not been defined. There is w which is the window size, so I think what is really meant is just window i. Here, the authors could help readers a little, by explicitly defining X_j and Y_j by their components which are the values of a particular AU at each of the time stamps in window j. It can be difficult to catch such small things which may confuse the reader when you know yourself what you mean, so I would suggest the authors to get a friendly review by a colleague who is unfamiliar with the method, and ask them to make a note of anything that is not immediately clear.

Thank you for your valuable comment. We have revised the manuscript so that the synchrony equation is introduced explicitly, with all indices and variables defined immediately following the equation.

p.9, l.228 & Eq. 3: "We concatenated each column (AUs) of S-matrix into a single vector v, referring to this as the flattened S-matrix." Here there is inconsistency between the quoted sentence and the notation used in Eq. 3. In the sentence an italic v is used, and in the equation boldface v_i and v_j are used. Also, I do not think it makes sense to say that each columns is concatenated. With what? It is simply that v_i and v_j are columns from the S-matrix, i.e. column vectors. On the other hand, if v is the concatenation of all of these column vectors, then it makes sense to call it the flattened S-matrix, but this does not seem to be used anywhere and only leads to confusion --- at least for me.

Thank you for this comment. We have revised the text and clarified Eq. 3 to remove the inconsistency. We now state explicitly that the entire S-matrix is flattened by concatenating all of its columns into a single vector $\mathbf{v}$. The notation has been made consistent, and $\mathbf{v}_{m}$and $\mathbf{v}_{n}$in Eq. 3 now clearly denote the flattened S-matrices of dyads $m$and $n$.

**Description of preprocessing**

The description of the preprocessing is not completely clear to me, so I hope these comments can help the authors improve the description of the preprocessing steps. The discussion of preprocessing is interrelated with the question of data quality and exclusion criteria. In section 3 Data, it is stated that 27 out of approximately 150 couples were selected for analysis due to missing or fragmented data "at the millisecond level." I believe that exluding such a large fraction of the data merits a more detailed description than the rather vague "due to missing or fragmented affective facial expression data at the millisecond level." First of all, I am not convinced that a single datum missing at the millisecond level would be a problem and second, I do not think that the data are even resolved at the millisecond level. Most commercial cameras record at 25, 30, or 60 fps corresponding to a time between frames of 40, 33, and 17 ms, respectively. So even at the highest framerate, the interval between samples is an order of magnitude above the millisecond level. On p.8, l.200-201 the authors say that 1000 samples is equivalent to 40 s, in other words the frame rate is 25 fps. This leaves me wondering what is meant by missing or fragmented data at the milisecond level. Including exact exclusion criteria in the manuscript and in the replication script would be a better way to describe the data exclusion.

Thank you for this helpful clarification. We now provide a detailed description of the preprocessing and exclusion criteria, including: (i) removal of uninformative frames in which all action units were zero, (ii) temporal synchronization of male and female partners by retaining only time points where both had valid data, and (iii) exclusion of dyads with insufficient overlapping synchronized data after preprocessing. These criteria explain why 27 dyads were retained from the original dataset and are now described explicitly in the manuscript and documented in the replication scripts.

In section 4.1 Preprocessing the initial comment that "Missing AU data occurred sparsely" (p.6, l.162) is imprecise. Please provide numbers, e.g, in % (mean and sd over the sample). Briefly thereafter the authors write "Additionally, there were moments when the data was [sic] unavailable for both partners simultaneously within the same dyad." (p. 7, l.164). I do not understand the difference between the two types of missing data. Is it just that sometimes there are data missing for only on partner and sometimes for both? And if so, why is this difference important?

Thank you for this comment. We have revised the manuscript to clarify the distinction and to provide quantitative information. We distinguish between individual-level missingness, where AU data are unavailable for one partner due to insufficient frame quality (e.g., occlusion or tracking failure), and dyad-level missingness, where AU data are unavailable for both partners simultaneously. Across the 27 dyads, individual-level non-informative data averaged 34.1% (SD = 18.3) for male participants and 24.6% (SD = 20.6) for female participants. Dyad-level missingness primarily resulted from concurrent individual-level tracking failures and is important because synchrony measures require valid data from both partners.

I fail to understand exactly why the authors treat the case when all AUs are zero as missing data. Is this a convention in the iMotions data format? And if so, how can this be distinguished from cases where all AUs are actually zero?

We appreciate this opportunity to clarify a technical nuance of the iMotions (AFFDEX) data stream. In automated FER, there is a fundamental distinction between a neutral (or non-emotive) face and a tracking failure. Empirically, the AFFDEX engine almost never produces a true zero value across all affect codes and action units simultaneously. Consequently, a string of absolute zeros across the entire vector is a known signature of a tracking dropout—typically caused by significant head rotation, occlusion (e.g., a hand or hat), or the participant moving out of the camera's frame. Treating these as zeros rather than missing would erroneously signal a state of perfect affective void, which would mathematically distort the synchrony estimates. We have added a brief clarification to the "Data Processing" section to make this distinction explicit.

I do not think Figure 1 is necessary to explain the simple concept of dropping rows with missing data. If kept, it should be converted into a table instead of a figure.

Thank you for this suggestion. We agree that Figure 1 is not necessary to explain this preprocessing step. Accordingly, Figure 1 has been removed from the revised manuscript.

The process of synchronizing the time stamps (p. 7, l.175) is not explained at all. Please provide an explanation. Perhaps because I do not understand the issue of synchronizing time stamps, I also fail to understand the decision "to use only the first minute of available data for each dyad." (p. 7, l. 180). This criterion along with the exclusion of most of the couples mean that only around 1.8% of the original dataset is used for the analysis. If the method does not handle "issues ... commmon in video analysis" (p. 7, l.165) it seems to be a major problem for the application of the method that the authors propose. I therefore urge the authors to think about how missing data can be handled in a more robust way. If the issue with dropping NA rows and then synchronizing the two individual time series in each couple is that the time series increasingly drift apart (or out-of-sync) then perhaps it is possible to take several 1-min epochs from the 10-minute conversations and align the two time series at the beginning of each epoch. If this is possible it would increase the data used in the analysis by up to a factor of 10.

Thank you for this comment. We have revised Section 4.1 to clarify the timestamp synchronization procedure and the rationale for using only the first minute of data. AU time series were extracted independently for each partner, and intermittent tracking failures resulted in misaligned timestamps and unequal numbers of valid frames. Synchronization was performed by aligning timestamps and retaining only time points with valid AU data for both partners, as required for zero-lag synchrony analysis.

The use of a one-minute segment reflects a limitation of the dataset rather than the proposed method. Most dyads provided approximately 60 s of continuous synchronized data after preprocessing, where the total synchronized duration across dyads had a mean of 113.3 s (SD = 65.9 s), making it difficult to reliably extract multiple independent one-minute epochs. We therefore selected the first continuous one-minute segment to ensure consistency across dyads. We agree that epoch-based re-synchronization is a promising extension and have now identified it as an important direction for future work.

Relevant baseline comparison

----------------------------

I think it is relevant to ask what the benefit is of extra detail, such as moment-to-moment synchrony as opposed to overall synchrony. In this case, where the dependent variable is a single measure (dichotomized QMI score) an approach using only average synchrony over the whole one-minute interval might be a more reasonable comparison. It would be interesting to know how such an approach (perhaps based on cross recurrence quantification analysis) compares to the moment-to-moment approach and to the current baseline approach using traditional statistical summary measures.

Thank you for this insightful comment. While moment-to-moment synchrony provides a richer characterization of interaction dynamics, we agree that comparisons with aggregate synchrony approaches (e.g., average synchrony over an interval or CRQA-based measures) are valuable. Given the limited continuous duration available in the current dataset, such comparisons could not be explored exhaustively and are therefore identified as important directions for future work

Including other approaches

--------------------------

This ties in with my previous comment about the relevant baseline comparison, viz. that other approaches have not been mentioned under related work. Specifically, the approach taken by the authors of using zero-lag cross-correlation has the implication that complementarity rather than similarity is not picked up by the method (see e.g. Dale et al. 2013). Others have analyzed facial expression dynamics in a way that takes such effects into account, e.g. Postma and Postma-Nilsenová (2016) and Marx et al. (2025). These are just two examples of papers that take such an approach using two different methods which are able to capture complementarity in addition to zero-lag synchrony. This type of literature seems to be completely ignored by the authors, and including and relating to this literature, e.g. in section 2.3 Characterization of Facial Synchrony would enable the reader to understand how the authors' approach differ.

Thank you for this helpful comment. We have revised Section 2.3 (Characterization of Facial Synchrony) to explicitly acknowledge and relate our work to prior approaches that model lagged and complementary coupling (e.g., Dale et al., 2013; Postma & Postma-Nilsenová, 2016; Marx et al., 2025). We clarify that the present study focuses on moment-to-moment zero-lag synchrony as a system-level baseline, and we discuss how this choice differs conceptually and methodologically from lag-based approaches. Despite this restriction, we show that time-localized zero-lag synchrony is sufficient to reveal clear separation between dyads in our dataset.

Figures are not publication quality

-----------------------------------

The quality of the figures in the PDF is low resolution, so I had to download each figure separately in order to be able to clearly see the details. This may be a problem with the journal's production system or it could be because the files are in a bitmap format (TIFF) rather than a vector format such as PDF.

Resolution issues aside, the figures all need some more work to make them publication quality. In many of the figures the text is so small that it is very hard to read, so the font size should be increased.

Rather than providing detailed feedback on each figure, I will do this for one figure and leave it to the authors to take similar actions for the remaining figures.

Below is detailed feedback on Figure 4.

- Organize the sub-plots by dichotomized QMI score (0 and 1) in two separate panels.
- Now the title (0 or 1) can be removed to give more space to the data.
- Remove all the common horizontal and vertical axes, except at the bottom and at the far left side. This frees up even more space and avoids redundancy.
- Add axes labels on the reamining axes and increase the font size.
- Consider a portrait layout instead of landscape to give a better aspect ratio for the individual sub-plots.
- Add a common color bar to indicate the scale (at top or bottom)
- Decrease the white space between the plots to give more space to the data.

Thank you for this helpful suggestion. We have updated the figures as suggested, considering the journal specifications for figures submission.

\## Minor comments

Since there are so many major comments that need to be addressed, it does not make sense to address all of the minor comments, so I will limit myself to a few (mostly recurring) issues.

* Equations: In many cases the equations are not inserted immediately after they are first referenced. This seems unconventional and makes it harder to read the manuscript (not much, but mainly it just flouts a convention).

Thank you for noting this. We have revised the manuscript to ensure that all equations are now placed immediately after their first reference in the text, improving readability and conforming to standard formatting conventions.

* Tables: Table 1 seems unnecessary, since the contents is well-known to any knowledgable reader. Tables 2 and 3 have horizontal rules between rows that should not be there.

Thank you for the comment. We have removed Table 1 from the manuscript, as its contents are well known to the target audience. In addition, the unnecessary horizontal rules between rows in Tables 2 and 3 have been removed to conform to standard table formatting guidelines.

* Number of digits: In some cases too many digits are provided, considering precision and sensitivity of the analysis. E.g., on p.11, l.273 and p.13, l.320 the baseline silhouette score is given as 0.4622. One or two significant digits would be sufficient here.

Thank you for the suggestion. We have reduced the number of reported digits and rounded values (e.g., silhouette scores) to an appropriate level of precision throughout the manuscript.

* Citing sources that are not open: I tried to access the link to the iMotions resource on facial expression analysis (reference 26), but was required to provide my personal information in order to obtain a copy. Consider if it is possible to cite an openly available and perhaps even peer-reviewed source instead.

We thank the reviewer for raising this important point regarding accessibility. We agree that citing openly available and peer-reviewed sources is valuable for transparency and reproducibility. We believe that the iMotions guidebook remains a useful reference, particularly for readers who have access to the software or who wish to understand its technical capabilities and implementation details; therefore, we have retained this citation.

To address the reviewer’s concern, we have additionally included an openly accessible, peer-reviewed study that explicitly documents the frame-by-frame facial expression analysis capability of the Affectiva engine integrated within iMotions. Specifically, we now cite:

Kulke L, Feyerabend D, Schacht A. A comparison of the Affectiva iMotions Facial Expression Analysis Software with EMG for identifying facial expressions of emotion. Frontiers in psychology. 2020;11:329.

**References:**

Bibby, E. S., McKinnon, A. M., Shaw, M., & Mattson, R. E. (2023). Accurately detecting happy facial expressions associates with perceptions of flirtatiousness. *Journal of Social and Personal Relationships*, *40*(4), 1288-1315.

Coan, J. A., & Sbarra, D. A. (2015). Social baseline theory: The social regulation of risk and effort. *Current opinion in psychology*, *1*, 87-91.

Karney, B. R., & Bradbury, T. N. (1995). The longitudinal course of marital quality and stability: A review of theory, methods, and research. *Psychological bulletin*, *118*(1), 3.

Woods, S. B., Priest, J. B., & Denton, W. H. (2013). A preliminary investigation of a telephone administration of the Quality of Marriage Index. *Journal of marital and family therapy*, *39*(2), 261-267.
