# Supplementary material for: Characterizing multidimensional facial synchrony for relationship quality detection
Source: PLoS One. 2026 Oct 1;21(10):e0356071. doi: 10.1371/journal.pone.0356071 (PMC13630251; doi:10.1371/journal.pone.0356071)
Supplement: Supplementary file 3 [file pone.0356071.s003.docx]

We thank the Reviewers for their careful reading of our manuscript and for their constructive and insightful comments. We have considered all comments and revised the manuscript accordingly.

For Reviewer 1, we provided point-by-point responses following the order of the comments. For Reviewer 2, we have organized our responses under the main headings, maintaining the same order and titles as presented in the review to facilitate clarity and ease of reference.

**Response to Reviewer 1**

**Point #1**

Thank you for this important comment. We have revised the manuscript to improve transparency in the exclusion process, and provide a clear summary of the number of dyads excluded at each stage, so that the derivation of the final sample is transparent.

**Point #2**

Thank you for this important comment. Figure 2 in the manuscript illustrates the dyads included in the analysis. Detailed information is provided in the code file *Part1_Check_Data.m*. Additionally, the README file has been updated to include more comprehensive definitions of the variables.

**Point #3**

Thank you for pointing this out, as it is an important aspect of the work that warrants clear explanation. We would like to clarify that the description of how missing data were handled was not removed, but rather revised and clarified in the previous version of the manuscript. As stated in Section 3.3, missing frames were excluded prior to the computation of cross-correlations and the construction of the S-matrix.

**Point #4**

Thank you for the suggestion. We have standardized the terminology by using *concurrent synchrony* throughout and removed “similarity-based synchrony” to improve clarity and alignment with the literature.

**Point #5**

Thank you for pointing this out. We replaced Figures 1, 4, 5, and 9 (in the previous revision) with higher-resolution versions to improve clarity. We have carefully worked on improving the image quality; however, it is possible that the remaining issues are related to the PDF generation process. If the figures are still not sufficiently clear, we would greatly appreciate your guidance, and we will address it immediately. We apologize for this issue.
In addition, we revised Figure 2 to reduce visual density in the dyad identifiers for better readability, and updated Figure 5 to capitalize the labels (Male, Female).

**Point #6**

Thank you for identifying these typos. Some of them were introduced during the track changes process, and all have now been addressed.

**Response to Reviewer 2**

**Major Comments**

**QMI Threshold**

Thank you for this thoughtful and important comment. We appreciate the opportunity to clarify the rationale and process underlying the selection of the QMI threshold.

First, we would like to clarify that the threshold of 80 was not chosen with the explicit goal of forcing an equal split of the sample. Rather, it emerged from a combination of data-driven inspection and theoretical considerations at the dyadic level.

From a data-driven perspective, we observed that the MDS representation of pairwise distances derived from the S-matrices revealed a natural separation into two clusters. When overlaid with summed QMI scores, dyads in one region were generally associated with lower scores, while those in the opposing region exhibited higher scores. This pattern suggested the presence of two qualitatively distinct groupings. In parallel, examination of the empirical distribution showed that the median summed QMI score was 79 in the analyzed subsample and 83 in the full sample of 150 couples. Therefore, a threshold near 80 aligns with the central tendency of both datasets, rather than being arbitrarily imposed.

Importantly, our intention was not to define a universal or transferable cutoff. We adopt a sample-specific threshold because the analysis is grounded in pairwise relational structure and unsupervised clustering, where labels are used post hoc for interpretation rather than imposed a priori. So the use of a sample-specific threshold is consistent with our analysis framework, where pairwise distance representations and unsupervised clustering define the underlying structure, and QMI-based grouping is applied post hoc to interpret this structure rather than to determine it.

We fully agree with the reviewer’s concern that reporting a threshold without context may lead to inappropriate reuse. For this reason, we have revised the manuscript to more explicitly describe the process used to arrive at this value. Specifically, the threshold reflects:

1. alignment with the empirical distribution, and
2. consistency with the structure observed in the unsupervised clustering space.

Finally, we emphasize that this threshold is sample-specific and was introduced to facilitate the sensitivity of the clustering analysis at the dyadic level, rather than to serve as a clinical or generalizable benchmark. We have now made this distinction more explicit in the revised manuscript to ensure that readers can understand and potentially adapt the methodology, rather than replicate the specific numeric value.

We thank the reviewer again for highlighting this point, as it has helped us improve the clarity and reproducibility of our work.

**Base line Comparison:**

We thank the reviewer for this important comment regarding the choice of baseline and feature comparison.

In this work, we adopted statistical feature extraction as a baseline, as it is a widely used approach in the literature. From a machine learning perspective, this corresponds to constructing feature representations based on summary statistics. We then compared the pairwise distance measurement based on these statistical features with the proposed multidimensional synchrony-based method.

A key consideration in this comparison is feature dimensionality. For the statistical approach, extracting $\boldsymbol{n}$statistical measures across 20 action units results in a feature space of size $\mathbf{20}\times\boldsymbol{n}$. Introducing windowing further increases this dimensionality, potentially leading to high-dimensional representations relative to the available data. In contrast, the zero-lag cross-correlation (synchrony-based) approach provides a more compact representation. Specifically, it produces one value per action unit per window, resulting in a feature space of $\mathbf{20}\times\mathbf{1}$ per window.

We acknowledge that one possible extension would be to apply window-based statistical analysis. However, this would significantly increase dimensionality and may not be suitable given the limited data duration (~60 seconds). Conversely, we evaluated a no-window version of the synchrony-based approach (as suggested by the reviewer), and obtained results consistent with the windowed analysis. The no-window synchrony-based analysis outperforms the statistical features but shows lower performance than the windowed cross-correlation approach.

The synchrony-based features demonstrated improved clustering performance compared to statistical features. This indicates that global, pairwise synchrony measurements capture meaningful structure in the data, even with fewer features.

Clustering quality was primarily assessed using the Silhouette score, which reflects both intra-cluster compactness and inter-cluster separation. To complement this, we reported the Euclidean distance between cluster centroids as an interpretable geometric measure of separation. Figure 6 was added to show the impact of the used configuration on the grouping pattern.

**Minor Comments**

**Add explanation for exclusion earlier**

Thank you for your comment. We have revised the sentence by adding the word “only” for clarity. In addition, we included a reference to the section where the data selection process is described in detail.

**Figure 2**

Thank you for this helpful suggestion. We have revised Figure 2 by sorting the dyads according to the length of the available synchronized data. This modification improves the readability of the figure and makes the comparison across dyads more intuitive.

**K-means clustering results**

Thank you for these insightful suggestions. We have further investigated both points to better understand the observed pattern.

First, regarding the use of MDS, we extended the analysis to a three-dimensional representation. The results showed a pattern consistent with the 2D projection. This suggests that the observed overlap is not due to the dimensionality reduction but rather reflects the underlying structure. This point has now been addressed in the revised manuscript.

Second, concerning the QMI threshold, we found that the misclassified cases are not all located near the threshold. This indicates that the misclassification cannot be explained solely by the dichotomization of QMI.

Thanks for the suggestion to include the QMI figure. We have added this figure in the current version, illustrating three different cases to show the impact of varying window sizes and to enable comparison with the no-window baseline.

**Report window sizes**

We thank the reviewer for this important clarification.

Our intention was to convey that the confusion matrices obtained across different window sizes were highly similar, indicating stable classification behavior. However, we agree that presenting a single confusion matrix does not demonstrate this consistency and that our original wording was unclear. We changed the wording in the manuscript to show that better.

In addition, we have updated all relevant figure captions to clearly specify the window size used.

**Practical implications and limitations**

We thank the reviewer for the thoughtful and constructive comments. Regarding the concern about clinical applicability, we agree that the current results do not provide sufficient evidence to support strong clinical claims. Our intention was not to suggest immediate clinical deployment, but rather to highlight a potential long-term direction. We have revised the manuscript to clarify that the current findings should be interpreted as proof-of-concept, demonstrating sensitivity of the proposed representation to dyadic interaction patterns, rather than as a clinically validated tool.

With respect to the suggestion of using continuous measures instead of dichotomization, we acknowledge this as an important limitation and have now explicitly stated it in the revised manuscript. However, given the small sample size (n = 27), our primary goal was to investigate whether the proposed representation can differentiate between qualitatively distinct dyadic states, rather than to model fine-grained variations in relationship quality.

In other words, the focus is on identifying whether meaningful structure exists in the interaction dynamics that aligns, even partially, with self-reported outcomes. Modeling QMI as a continuous variable would require a larger dataset to ensure robustness and avoid overfitting, which is beyond the scope of the current work.

Regarding the confusion matrix and the observed false positive rate for non-stressed couples, we agree that performance is not uniform across groups. The purpose of the confusion matrix in this study is not to frame the approach as a classification model, but rather to provide an external evaluation of the clustering results and a general way of assessing how dyads are categorized. It serves to illustrate the agreement between the cluster assignments and the true labels. Although both the baseline and the synchrony-based methods exhibit similar false positive rates, the distribution of dyads in the space is more clearly structured when using the synchrony-based representation.

**Other minor issues**

- Thank you for pointing this out. We have corrected the notation to ensure that a proper mathematical minus sign is used instead of a hyphen in the sentence following Eq. 6.

Figure 5:

- Thank you for the clarification. We have revised the caption of Figure 5 to replace “x-axis” and “y-axis” with “horizontal axis” and “vertical axis,” ensuring more precise and appropriate terminology.
- Thank you for the observation. We have removed the references to “(A)” and “(B)” from the caption and revised it to describe the tree icons representing the two classes, ensuring alignment with the figure.
