# Supplementary material for: Characterizing multidimensional facial synchrony for relationship quality detection
Source: PLoS One. 2026 Oct 1;21(10):e0356071. doi: 10.1371/journal.pone.0356071 (PMC13630251; doi:10.1371/journal.pone.0356071)
Supplement: Supplementary file 4 [file pone.0356071.s004.docx]

**We thank the reviewers for their valuable comments. Below, we provide point-by-point responses to each comment. Reviewer comments are presented first, followed by our responses (highlighted in red for illustration purposes).**

Reviewer #1: While exclusions due to technical limitations are expected in this type of data, the magnitude of this reduction is substantial and requires clear justification. Despite the revisions, the exclusion criteria remain insufficiently specified. Terms such as “insufficient data” and “substantial portions of unusable data” are used, but no explicit quantitative thresholds are provided. At this stage, it is essential that the authors clearly define how much missing or unusable data led to exclusion (e.g., proportion of missing frames, minimum duration of valid data). Without such thresholds, the exclusion process cannot be evaluated or reproduced. In addition, the distinction between “insufficient data” and “substantial portions of unusable data” remains unclear. As written, these appear to reflect overlapping concepts, and it is not possible to determine how decisions were made at each stage of the pipeline.
More importantly, the manuscript does not provide sufficient justification for the extent of data reduction. It remains unclear whether the exclusion process necessarily leads to a final sample of 27 dyads, or whether a larger portion of the dataset could have been retained under clearly defined criteria. Given the size of the original dataset, this point requires direct empirical support. The authors should evaluate whether alternative, less restrictive inclusion strategies (e.g., segment-level inclusion or different missing-data thresholds) would increase the usable sample size. If not, this should be demonstrated and explained fully to provide clearer justification for the exclusion pipeline.

We thank the reviewer for this important comment. We agree that the reduction from the original dataset to the final sample of 27 dyads requires clear justification. The dataset used in this study is a pre-existing dataset collected at our institution and was not originally optimized for the purpose of automated facial synchrony analysis. Rather, this present study extends our prior work linking discrete iMotions-detected facial affect events with relationship quality by applying automated facial expression analysis and synchrony measures to investigate dyadic relationship dynamics. To our knowledge, open-source datasets containing dyadic facial-expression recordings from married couple interaction together with relationship quality labels are largely unavailable, likely in part because facial video constitutes sensitive personal data. Given the scarcity of suitable public datasets and our prior work using this dataset, we believe it was reasonable to utilize this dataset for the present synchrony work.

The large number of exclusions primarily reflects limitations in the quality and availability of the facial recordings rather than the application of highly restrictive selection criteria. After processing the videos with iMotions/AFFDEX, many recordings contained substantial periods in which facial landmarks could not be reliably tracked, resulting in frames with all AU values equal to zero (e.g., participants leaning out of frame, turning away from the camera, or briefly occluding their faces with their hands). We treated these frames as non-informative and excluded them from analysis. Although missing-data approaches such as interpolation could be considered, the amount of missing data in many recordings was substantial. Because our goal was to quantify facial synchrony using genuine observed facial behavior, we chose a conservative approach and restricted analyses to valid tracked frames rather than generating synthetic facial-expression values through interpolation.

The primary inclusion criterion was the availability of overlapping valid facial-expression data from both partners within a dyad. We examined the duration of valid overlapping recordings and observed that most retained dyads contained approximately one minute of valid data. Requiring substantially longer durations would have further reduced the sample size, whereas allowing much shorter durations would have increased the sample size at the expense of data quality. Therefore, rather than maximizing the number of retained dyads, we prioritized the availability of sufficiently long periods of valid facial-expression recordings from both partners, balancing sample size against data quality.

To clarify our terminology, "insufficient data" refers to cases in which the original recordings were unavailable, heavily occluded, or contained extensive periods of tracking failure, resulting in little or no usable facial-expression information. In contrast, "substantial data loss" refers to dyads that, after removal of non-informative frames, contained less than the minimum duration threshold required for analysis. We selected a threshold of 60 seconds because it represented a reasonable compromise between retaining an adequate sample size and ensuring sufficient data availability for reliable synchrony estimation. We have revised the manuscript to clarify these distinctions, differentiate between missing data and insufficient overlapping valid recordings, and provide a more transparent description of the exclusion process and its rationale.

Reviewer 2

1. In their response letter the authors write that "we evaluated a no-window version of the synchrony-based approach (as suggested by the reviewer), and
   obtained results consistent with the windowed analysis." This suggests that an aggregated approach is almost as good as the windowed approach, but in the abstract they highlight a "sliding-window framework that computes moment-to-moment facial synchrony." It would seem that the sliding window approach is not so crucial, which would also be surprising, given a window size that is 2/3 of the entire time series. So, in their description in the abstract of "moment-to-moment synchrony" I suggest the authors tone down the emphasis on "evolving synchrony state of each dyad over time" and focus on the synchrony. It seems to be the synchrony rather than the windowed analysis that does the heavy lifting vis-a-vis the results, and it would be a more honest description if the abstract reflected this. I like the new paragraphs discussing this is the Results and Discussion section, so there is not need to change the manuscript itself, just the abstract.

We thank the reviewer for this thoughtful observation. In light of this comment, we revised the abstract to reduce emphasis on moment-to-moment dynamics and instead focus on the synchrony representation and its association with marital relationship quality.

2. In a newly added paragraph about the exclusion of data the authors write: "This initial exclusion did not account for additional quality issues, such as lack of synchronization" and "41 were further excluded due to either substantial portions of unusable data or poor synchronization between male and female participants." This is very confusing to me, since synchronization could be understood to refer to the synchrony measure. But I am almost certain the authors mean something else here, and this has to be clarified.

We thank the reviewer for highlighting this ambiguity. We agree that the term *synchronization* could be misinterpreted as referring to the facial synchrony measure analyzed in this study. In this context, however, *synchronization* simply refers to the temporal alignment of the recordings from the male and female participants within a dyad (aka whether two datapoints from both participants are both available at the same timestamp), rather than the behavioral synchrony computed from facial action units. To avoid confusion, we have replaced the term synchronization with temporal alignment in the manuscript.
