# Supplementary material for: Characterizing multidimensional facial synchrony for relationship quality detection
Source: PLoS One. 2026 Oct 1;21(10):e0356071. doi: 10.1371/journal.pone.0356071 (PMC13630251; doi:10.1371/journal.pone.0356071)
Supplement: Peer review history

# Peer review: Decision Letter 0

**DOI:** 10.1371/journal.pone.0356071.r001

**Authors:** Sebastian Wallot

25 Nov 2025

Dear Dr. Fora,

- **When revising the manuscript, pay particular attention toward situating our work properly in the literature, providing sufficient detail on all steps involved in the data preprocessing and analysis process, an ensure that the data you make available are properly formatted and labeled and can be readily understood by third parties. A replication script will greatly help other researches to follow your analysis and make sense of the data. For course, your response should not be limited to these points. The two reviewers list a number of major and minor issues, all of which should be addressed in a revision.**

plosone@plos.org. When you're ready to submit your revision, log on to https://www.editorialmanager.com/pone/ and select the 'Submissions Needing Revision' folder to locate your manuscript file.

- A rebuttal letter that responds to each point raised by the academic editor and reviewer(s). You should upload this letter as a separate file labeled 'Response to Reviewers'.
- A marked-up copy of your manuscript that highlights changes made to the original version. You should upload this as a separate file labeled 'Revised Manuscript with Track Changes'.
- An unmarked version of your revised paper without tracked changes. You should upload this as a separate file labeled 'Manuscript'.

If you would like to make changes to your financial disclosure, please include your updated statement in your cover letter. Guidelines for resubmitting your figure files are available below the reviewer comments at the end of this letter.

If applicable, we recommend that you deposit your laboratory protocols in protocols.io to enhance the reproducibility of your results. Protocols.io assigns your protocol its own identifier (DOI) so that it can be cited independently in the future. For instructions see: https://journals.plos.org/plosone/s/submission-guidelines#loc-laboratory-protocols. Additionally, PLOS ONE offers an option for publishing peer-reviewed Lab Protocol articles, which describe protocols hosted on protocols.io. Read more information on sharing protocols at https://plos.org/protocols?utm_medium=editorial-email&utm_source=authorletters&utm_campaign=protocols.

We look forward to receiving your revised manuscript.

Kind regards,

Sebastian Wallot, Ph.D

Academic Editor

PLOS ONE

**Journal Requirements:**

1. When submitting your revision, we need you to address these additional requirements.

Please ensure that your manuscript meets PLOS ONE's style requirements, including those for file naming. The PLOS ONE style templates can be found at

https://journals.plos.org/plosone/s/file?id=wjVg/PLOSOne_formatting_sample_main_body.pdf and

https://journals.plos.org/plosone/s/file?id=ba62/PLOSOne_formatting_sample_title_authors_affiliations.pdf

2. We note that your Data Availability Statement is currently as follows: All relevant data are within the manuscript and its Supporting Information files.

Please confirm at this time whether or not your submission contains all raw data required to replicate the results of your study. Authors must share the “minimal data set” for their submission. PLOS defines the minimal data set to consist of the data required to replicate all study findings reported in the article, as well as related metadata and methods (https://journals.plos.org/plosone/s/data-availability#loc-minimal-data-set-definition).

For example, authors should submit the following data:

- The values behind the means, standard deviations and other measures reported;

- The values used to build graphs;

- The points extracted from images for analysis.

Authors do not need to submit their entire data set if only a portion of the data was used in the reported study.

If your submission does not contain these data, please either upload them as Supporting Information files or deposit them to a stable, public repository and provide us with the relevant URLs, DOIs, or accession numbers. For a list of recommended repositories, please see https://journals.plos.org/plosone/s/recommended-repositories.

If there are ethical or legal restrictions on sharing a de-identified data set, please explain them in detail (e.g., data contain potentially sensitive information, data are owned by a third-party organization, etc.) and who has imposed them (e.g., an ethics committee). Please also provide contact information for a data access committee, ethics committee, or other institutional body to which data requests may be sent. If data are owned by a third party, please indicate how others may request data access.

3. Your ethics statement should only appear in the Methods section of your manuscript. If your ethics statement is written in any section besides the Methods, please move it to the Methods section and delete it from any other section. Please ensure that your ethics statement is included in your manuscript, as the ethics statement entered into the online submission form will not be published alongside your manuscript.

4. If the reviewer comments include a recommendation to cite specific previously published works, please review and evaluate these publications to determine whether they are relevant and should be cited. There is no requirement to cite these works unless the editor has indicated otherwise.

Reviewers' comments:

**Comments to the Author**

1. Is the manuscript technically sound, and do the data support the conclusions?

Reviewer #1: Yes

Reviewer #2: Partly

**********

2. Has the statistical analysis been performed appropriately and rigorously?

Reviewer #1: Yes

Reviewer #2: N/A

**********

3. Have the authors made all data underlying the findings in their manuscript fully available?

The PLOS Data policy

Reviewer #1: No

Reviewer #2: No

**********

4. Is the manuscript presented in an intelligible fashion and written in standard English?

Reviewer #1: Yes

Reviewer #2: Yes

**********

**Reviewer #1:**  This manuscript tackles a fascinating and timely question about how moment-to moment facial synchrony can be used to characterize relationship quality. It is encouraging to see this type of dynamic, data-driven approach applied to real interpersonal processes, as such work advances the field substantially. I found the effort commendable and promising, though several points would benefit from clarification and refinement:

1. Actual numerical results are reported in the Introduction (line 32). Numerical findings should not appear in this section.

2. The claim that this is “the first study to characterize moment-to-moment synchrony in facial expressions for relationship quality detection using such a comprehensive and unsupervised approach” (lines 33–35) is overstated. Prior studies have already examined naturally occurring facial synchrony as an indicator of relationship quality in various interpersonal contexts, including Machluf-Ruttner et al. (2024), Rohr et al. (2022), Yokotani et al. (2020), and Cheong et al. (2023). The claim of novelty should be moderated accordingly.

3. The term “Action Unit (AU)” is abbreviated at the start of the introduction (line 25) and used throughout the manuscript. When used as part of the text, it should appear in full as “action unit,” whereas in technical contexts referring to specific units (e.g., AU1, AU2), the abbreviation is more appropriate.

4. Typo on line 213: extra space before the period in “...expressions over time .”.

5. Figures require revision:

- Figure 1 may be unnecessary given the clarity of the textual explanation.

- The title of Figure 2 uses an internal label that is inappropriate for publication.

- Image quality across figures is poor and should be improved through direct export rather than screenshots.

- Figures 2, 3, and 8 show only a single dyad; if possible, data from the full sample should be represented.

- Axis and legend labels should be consistently capitalized.

- Some figures lack sufficient explanatory detail in their captions (e.g., Figure 7).

6. The manuscript places heavy emphasis on methodological considerations, such as windowing and clustering, while providing limited discussion of the conceptual implications. The theoretical meaning of linking facial synchrony to relationship quality in this way could be developed more clearly and prominently.

**Reviewer #2:**  # Review

## Summary

The article "Characterizing Multidimensional Facial Synchrony for Relationship

Quality Detection" presents a new way to analyze facial expressions in dyads

and the method is applied to an existing dataset based on video recordings of

27 heterosexual couples engaged in discussion in a lab setting.

The main contribution of the research is that facial expressions in dyads are

analyzed as a moment-by-moment process measure rather than averaged over larger

time intervals or by using other summary measures where the detailed

time-evolution is lost.

The potential of the method is an easy, cheap and non-intrusive way (relative

to say EMG) to use facial expression analysis based on video recordings to

assess the temporal dynamics of facial expression synchrony. This potential

alone merits its consideration for publication and it can conceivably be used

for many other applications than assessing relationship quality.

I appreciate the authors' efforts and it was interesting to read the paper,

albeit at times it required more effort than I think should be asked of a

curious reader. So while there are many things to like about the manuscript it

also has quite a few major issues. My assessment is therefore that it will take

a major revision to address these issues, but I also believe that if the

authors can address all of the major issues successfully it will lead to a

significant improvement of the paper.

My major comments and a few minor comments follow below.

## Major comments

Replicability

--------------

I commend the authors for making the full dataset available. I downloaded the

set of files provided in the hope that I could also replicate the results in

the article by following the description in the methods section. However, I

quickly realized that (as far as I can tell) there was no information about how

the female and male participants were matched in couples. A guess is that Dyad

= Respondent - Gender, but this leaves about a dozen unmatched participants, so

I am not certain.

The remedy for this is simple: The authors should provide one or more

replication scripts with the code for the analysis performed in the paper. This

can be in any software the authors have used such as R, Python, Julia,

Octave/MATLAB, etc. Please also ensure that both data and replication scripts

are available in a public repository such as Zenodo, Open Science Framework,

figshare or similar.

Dichotomizing the variables

---------------------------

The QMI score is dichtomized by what appears to be a median split (although

this is not explicitly stated). The description on p.6, l.142-146 indicates

that the threshold of 80 was selected to obtain an even split. If this was the

reason, please write this explicitly, or provide an alternative reason.

More fundamentally, dichotomizing the dependent variable like this discards

data and variation within the data that is then no longer available in the

analysis. I suspect this was done to simplify the analysis, but it would be

interesting to see results of an analysis where this information was retained,

e.g. a multiple linear regression analysis with the (un-dichotomized)

dyad-level QMI.

Similarly the independent variable is dichotomized by requiring two clusters in

the k-means clustering step. There are alternatives, such as logistic

regression, where the independent variable can be retained, even if the

dependent variable is dichotomized. It would be instructive to see results of

an analysis where neither the independent nor the dependent variable have been

dichotomized. There may be good reasons to do this, but it should not be done

as the first step and the reasons should be provided to the reader.

Motivation for the method

--------------------------

In brief, the method takes simultaneous videorecordings of individuals in a

couple and use an automated algorithm to extract AU activation for each AU and

each frame of the video. This gives a time series of AU activations that can be

matched up for the two individuals in each dyad. These are used to construct a

so-called S-matrix of zero-lag correlations between AUs (rows) in a speficic

time window, and time windows (columns) in a sliding window approach where the

window of width w, is moved by some step size along the time series. In a next

step pairwise Euclidean distances between vectors that are columns of the

S-matrix are calculated and subjugated to multidimensional scaling (MDS) to

obtain a two-dimensional representation. The data points in this

two-dimensional space are then used as input to a k-means clustering algorithm

fixed at producing two clusters from the data. The cluster membership of each

couple is then compared to the dichotomized quality of marriage index (QMI)

categorizing couples as either stressed or non-stressed.

There are several places along the way, when I read the description of the

method summarized above, where I think a little motivation would help to

understand the method. I constantly kept wondering why the authors made some

very specific choices where there were other alternatives I could think of. I

do not ask the authors to consider such alternatives in detail, but merely

to provide some reasoning for the steps in the analysis. A figure explaining

the method in a conceptual way could go a long way to increase the readability

of the manuscript, e.g. along the lines of Figure 1 in Shadeydeh et al. (2021).

Some of the choices I would like to see explained are:

i) What is the purpose of calculating the pairwise distances bewteen the

columns of the S-matrix?

ii) why do the authors use MDS to perform dimensional reduction when k-means

clustering is fully capable of operating on higher-dimensional data.

iii) I also wonder what would happen if a different number of clusters had been

found. I understand that the authors are interested in having two clusters to be

able to compare to the dichotomized QMI score, but at least a consideration of

the number of clusters seems appropriate to include.

Description of the method

-------------------------

Apart from the reasoning behind the method, I would also like to see it

described a bit more concisely. In several places I had to stop and think about

exactly what was meant by a particular phrase or expression. Here are a few

examples:

p.8, l.198: "where X_j and Y_j represents the AU vectors of the two individuals

within the window w_i." This makes sense, but it is the first time the reader

encounters X_j and Y_j and w_i has not been defined. There is w which is the

window size, so I think what is really meant is just window i. Here, the

authors could help readers a little, by explicitly defining X_j and Y_j by

their components which are the values of a particular AU at each of the time

stamps in window j. It can be difficult to catch such small things which may

confuse the reader when you know yourself what you mean, so I would suggest

the authors to get a friendly review by a colleague who is unfamiliar with the

method, and ask them to make a note of anything that is not immediately clear.

p.9, l.228 & Eq. 3: "We concatenated each column (AUs) of S-matrix into a

single vector v, referring to this as the flattened S-matrix." Here there is

inconsistency between the quoted sentence and the notation used in Eq. 3. In

the sentence an italic v is used, and in the equation boldface v_i and v_j are

used. Also, I do not think it makes sense to say that each columns is

concatenated. With what? It is simply that v_i and v_j are columns from the

S-matrix, i.e. column vectors. On the other hand, if v is the concatenation of

all of these column vectors, then it makes sense to call it the flattened

S-matrix, but this does not seem to be used anywhere and only leads to

confusion --- at least for me.

Description of preprocessing

----------------------------

The description of the preprocessing is not completely clear to me, so I hope

these comments can help the authors improve the description of the

preprocessing steps. The discussion of preprocessing is interrelated with the

question of data quality and exclusion criteria. In section 3 Data, it is

stated that 27 out of approximately 150 couples were selected for analysis due

to missing or fragmented data "at the millisecond level." I believe that

exluding such a large fraction of the data merits a more detailed description

than the rather vague "due to missing or fragmented affective facial expression

data at the millisecond level." First of all, I am not convinced that a single

datum missing at the millisecond level would be a problem and second, I do not

think that the data are even resolved at the millisecond level. Most

commercial cameras record at 25, 30, or 60 fps corresponding to a time between

frames of 40, 33, and 17 ms, respectively. So even at the highest framerate,

the interval between samples is an order of magnitude above the millisecond

level. On p.8, l.200-201 the authors say that 1000 samples is equivalent to 40

s, in other words the frame rate is 25 fps. This leaves me wondering what

is meant by missing or fragmented data at the milisecond level. Including exact

exclusion criteria in the manuscript and in the replication script would be a

better way to describe the data exclusion.

In section 4.1 Preprocessing the initial comment that "Missing AU data ocurred

sparsely" (p.6, l.162) is imprecise. Please provide numbers, e.g, in % (mean

and sd over the sample). Briefly thereafter the authors write "Additionally,

there were moments when the data was [sic] unavailable for both partners

simultaneously within the same dyad." (p. 7, l.164). I do not understand the

difference between the two types of missing data. Is it just that sometimes

there are data missing for only on partner and sometimes for both? And if so,

why is this difference important?

I fail to understand exactly why the authors treat the case when all AUs are

zero as missing data. Is this a convention in the iMotions data format? And if

so, how can this be distinguished from cases where all AUs are actually zero?

I do not think Figure 1 is necessary to explain the simple concept of dropping

rows with missing data. If kept, it should be converted into a table instead of

a figure.

The process of synchronizing the time stamps (p. 7, l.175) is not explained at

all. Please provide an explanation.

Perhaps because I do not understand the issue of synchronizing time stamps, I

also fail to understand the decision "to use only the first minute of available

data for each dyad." (p. 7, l. 180). This criterion along with the exclusion of

most of the couples mean that only around 1.8% of the original dataset is used

for the analysis. If the method does not handle "issues ... commmon in video

analysis" (p. 7, l.165) it seems to be a major problem for the application of

the method that the authors propose. I therefore urge the authors to think

about how missing data can be handled in a more robust way. If the issue with

dropping NA rows and then synchronizing the two individual time series in each

couple is that the time series increasingly drift apart (or out-of-sync) then

perhaps it is possible to take several 1-min epochs from the 10-minute

conversations and align the two time series at the beginning of each epoch. If

this is possible it would increase the data used in the analysis by up to a

factor of 10.

Relevant baseline comparison

----------------------------

I think it is relevant to ask what the benefit is of extra detail, such as

moment-to-moment synchrony as opposed to overall synchrony. In this case, where

the dependent variable is a single measure (dichotomized QMI score) an approach

using only average synchrony over the whole one-minute interval might be a more

reasonable comparison. It would be interesting to know how such an approach

(perhaps based on cross recurrence quantification analysis) compares to the

moment-to-moment approach and to the current baseline approach using

traditional statistical summary measures.

Including other approaches

--------------------------

This ties in with my previous comment about the relevant baseline comparison,

viz. that other approaches have not been mentioned under related work.

Specifically, the approach taken by the authors of using zero-lag

cross-correlation has the implication that complementarity rather than

similarity is not picked up by the method (see e.g. Dale et al. 2013). Others

have analyzed facial expression dynamics in a way that takes such effects into

account, e.g. Postma and Postma-Nilsenová (2016) and Marx et al. (2025). These

are just two examples of papers that take such an approach using two different

methods which are able to capture complementarity in addition to zero-lag

synchrony. This type of literature seems to be completely ignored by the

authors, and including and relating to this literature, e.g. in section 2.3

Characterization of Facial Synchrony would enable to reader to understand how

the authors' approach differ.

Figures are not publication quality

-----------------------------------

The quality of the figures in the PDF is low resolution, so I had to download

each figure separately in order to be able to clearly see the details. This may

be a problem with the journal's production system or it could be because the

files are in a bitmap format (TIFF) rather than a vector format such as PDF.

Resolution issues aside, the figures all need some more work to make them

publication quality. In many of the figures the text is so small that it is

very hard to read, so the font size should be increased.

Rather than providing detailed feedback on each figure, I will do this for one

figure and leave it to the authors to take similar actions for the remaining

figures.

Below is detailed feedback on Figure 4.

* Organize the sub-plots by dichotomized QMI score (0 and 1) in two separate

panels.

* Now the title (0 or 1) can be removed to give more space to the data.

* Remove all the common horizontal and vertical axes, except at the bottom and

at the far left side. This frees up even more space and avoids redundancy.

* Add axes labels on the reamining axes and increase the font size.

* Consider a portrait layout instead of landscape to give a better aspect ratio

for the individual sub-plots.

* Add a common color bar to indicate the scale (at top or bottom)

* Decrease the white space between the plots to give more space to the data.

## Minor comments

Since there are so many major comments that need to be addressed, it does not

make sense to address all of the minor comments, so I will limit myself to a

few (mostly recurring) issues.

* Equations: In many cases the equations are not inserted immediately after

they are first referenced. This seems unconventional and makes it harder to

read the manuscript (not much, but mainly it just flouts a convention).

* Tables: Table 1 seems unnecessary, since the contents is well-known to any

knowledgable reader. Tables 2 and 3 have horizontal rules between rows that

should not be there.

* Number of digits: In some cases too many digits are provided, considering

precision and sensitivity of the analysis. E.g., on p.11, l.273 and p.13,

l.320 the baseline silhouette score is given as 0.4622. One or two

significant digits would be sufficient here.

* Citing sources that are not open: I tried to access the link to the

iMotions resource on facial expression analysis (reference 26), but was

required to provide my personal information in order to obtain a copy.

Consider if it is possible to cite an openly available and perhaps even

peer-reviewed source instead.

## References

Dale, Rick, et al. "Beyond synchrony: Complementarity and asynchrony in joint

action." Proceedings of the Annual Meeting of the Cognitive Science Society.

Vol. 35. No. 35. 2013.

Marx, Anton KG, et al. "Emotional contagion in dyadic online video

conferences—empirical evidence based on self-report and facial expression

data." Frontiers in Psychology 16 (2025): 1546303.

Shadaydeh, Maha, et al. "Analyzing the direction of emotional influence in

nonverbal dyadic communication: A facial-expression study." IEEE Access 9

(2021): 73780-73790.

Postma, Eric O., and Marie Postma-Nilsenová. "Measuring the Causal Dynamics of

Facial Interaction with Convergent Cross Mapping." CogSci. 2016.

**********

what does this mean?). If published, this will include your full peer review and any attached files.

If you choose “no”, your identity will remain anonymous but your review may still be made public.

**Do you want your identity to be public for this peer review?**  For information about this choice, including consent withdrawal, please see our Privacy Policy

Reviewer #1: No

Reviewer #2: **Yes:**  Dan Mønster

**********

[NOTE: If reviewer comments were submitted as an attachment file, they will be attached to this email and accessible via the submission site. Please log into your account, locate the manuscript record, and check for the action link "View Attachments". If this link does not appear, there are no attachment files.]

To ensure your figures meet our technical requirements, please review our figure guidelines: https://journals.plos.org/plosone/s/figures

You may also use PLOS’s free figure tool, NAAS, to help you prepare publication quality figures: https://journals.plos.org/plosone/s/figures#loc-tools-for-figure-preparation.

NAAS will assess whether your figures meet our technical requirements by comparing each figure against our figure specifications.

# Author comment: Author response to Decision Letter 1

**DOI:** 10.1371/journal.pone.0356071.r002

23 Jan 2026

We thank the Editor and the two Reviewers for their careful reading of our manuscript and for their constructive and insightful comments. We have carefully considered the Editor’s comments as well as those of both Reviewers, and we have revised the manuscript accordingly. Below, we reproduce each reviewer’s comment followed by our detailed response. For ease of tracking, all responses are shown in red.

In response to the reviewers’ request for improved reproducibility, we have attached the full analysis code as supplementary material. All processed datasets are provided in MATLAB .mat files, and the complete preprocessing and analysis pipeline is implemented in MATLAB .m files. These scripts serve as replication code and allow third parties to reproduce the results reported in the manuscript. The source data files in their original format (.csv) are archived and publicly available on Zenodo to ensure long-term accessibility and transparency. Both the source data and the analysis code are provided via two separate url links.

Review #1

1. Actual numerical results are reported in the Introduction (line 32). Numerical findings should not appear in this section.

Thank you for this comment. We have revised the manuscript accordingly and removed numerical findings from the Introduction.

2. The claim that this is “the first study to characterize moment-to-moment synchrony in facial expressions for relationship quality detection using such a comprehensive and unsupervised approach” (lines 33–35) is overstated. Prior studies have already examined naturally occurring facial synchrony as an indicator of relationship quality in various interpersonal contexts, including Machluf-Ruttner et al. (2024), Rohr et al. (2022), Yokotani et al. (2020), and Cheong et al. (2023). The claim of novelty should be moderated accordingly.

We thank the reviewer for pointing out that the original novelty claim was overstated and for referencing relevant prior work on facial synchrony in interpersonal and relational contexts. We agree with this assessment and have revised the manuscript accordingly. The novelty statement has been rewritten to emphasize that the contribution of this work lies in introducing a fully automated, unsupervised machine-learning framework for marital relationship quality detection based on dynamic, moment-to-moment facial synchrony, rather than in being the first to study facial synchrony per se.

3. The term “Action Unit (AU)” is abbreviated at the start of the introduction (line 25) and used throughout the manuscript. When used as part of the text, it should appear in full as “action unit,” whereas in technical contexts referring to specific units (e.g., AU1, AU2), the abbreviation is more appropriate.

Thank you for this helpful clarification. We have made edits accordingly throughout the manuscript.

4. Typo on line 213: extra space before the period in “...expressions over time .”.

Thank you for the comment. We have fixed the typo.

5. Figures require revision:

- Figure 1 may be unnecessary given the clarity of the textual explanation.

Thank you for this comment. We agree with this assessment and we have removed Figure 1.

- The title of Figure 2 uses an internal label that is inappropriate for publication.

Thank you for this comment. We have removed the internal label and made everything clear in the caption.

- Image quality across figures is poor and should be improved through direct export rather than screenshots.

Thank you for this comment. We have updated all figures to comply with the journal’s figure specifications.

- Figures 2, 3, and 8 show only a single dyad; if possible, data from the full sample should be represented.

We thank the reviewer for this valuable suggestion. Figures 2, 3, and 8 (as numbered in the previous version) present results from a single representative dyad and are included for illustrative purposes, enabling a clear and interpretable demonstration of the proposed methodology. To ensure transparency and reproducibility, data from all dyads are publicly available for interested readers. In addition, Figure 5 (formerly Figure 4) presents synchrony profiles for the full sample, thereby capturing the overall patterns observed across all dyads and demonstrating the consistency of the proposed approach at the group level.

- Axis and legend labels should be consistently capitalized.

Thank you for this comment. We have capitalized all axis and legend labels.

- Some figures lack sufficient explanatory detail in their captions (e.g., Figure 7).

Thank you for this comment. We have provided more detail in the caption.

6. The manuscript places heavy emphasis on methodological considerations, such as windowing and clustering, while providing limited discussion of the conceptual implications. The theoretical meaning of linking facial synchrony to relationship quality in this way could be developed more clearly and prominently.

We thank the reviewer for this insightful critique. We agree that while the technical advancements of the S-matrix approach are significant, its value is ultimately realized through its ability to illuminate the psychological processes underlying marital health. In the revised manuscript, we have significantly expanded the theoretical framework in the Introduction and Discussion sections. Specifically, we now frame facial synchrony not merely as a correlate of satisfaction, but as a dynamic marker of interpersonal attunement and communication flexibility. We have also added practical implications. More specifically, we now discuss the potential for this method to serve as an objective digital biomarker in clinical settings. These additions help bridge the gap between our systems-science methodology and the interpersonal-science theory it supports.

Reviewer #2

Replicability

I commend the authors for making the full dataset available. I downloaded the set of files provided in the hope that I could also replicate the results in the article by following the description in the methods section. However, I quickly realized that (as far as I can tell) there was no information about how the female and male participants were matched in couples. A guess is that Dyad = Respondent - Gender, but this leaves about a dozen unmatched participants, so I am not certain.

The remedy for this is simple: The authors should provide one or more replication scripts with the code for the analysis performed in the paper. This can be in any software the authors have used such as R, Python, Julia, Octave/MATLAB, etc. Please also ensure that both data and replication scripts are available in a public repository such as Zenodo, Open Science Framework, figshare or similar.

We thank the reviewer for this comment and apologize for the lack of clarity regarding dyad–partner matching. We have now provided a self-report table that explicitly lists the dyad ID, and the corresponding individual QMI and other self-report scores, making the couple matching unambiguous.

We have also included replication scripts with all required data as supplementary materials for the review process. The Zenodo repository currently hosts the original dataset for general reuse; we will release an updated version including the dyad-mapping file and replication code, or archive these materials with the final supplementary files upon publication.

We appreciate the reviewer’s suggestion, which helped strengthen the reproducibility of this work.

Dichotomizing the variables

The QMI score is dichtomized by what appears to be a median split (although this is not explicitly stated). The description on p.6, l.142-146 indicates that the threshold of 80 was selected to obtain an even split. If this was the reason, please write this explicitly, or provide an alternative reason.

We thank the reviewer for identifying the need for a more explicit rationale regarding our scoring threshold. The choice of a dyad-level threshold of 80 (equivalent to an average individual score of 40) was informed by both the distributional properties of our community sample and our theoretical focus on the dyad as the primary unit of analysis. While clinical benchmarks – such as the score of greater than or equal to 29 suggested by Woods et al. (2013) – are useful for identifying severe individual distress, community samples of married couples are characteristically skewed toward higher satisfaction due to marital selection effects—where highly distressed couples exit the population via separation or divorce.

In our sample, a strictly clinical cutoff would have resulted in an extremely unbalanced partition, failing to capture the "at-risk" or "moderately dissatisfied" couples who still exhibit meaningful variance in facial synchrony. By using a threshold of 80, we partitioned the sample into "Satisfied" versus "Distressed/At-Risk" dyads. This approach provides the clustering algorithm with enough signal to distinguish between highly adjusted systems and those experiencing relational strain, which is more appropriate for a community-based detection model. We have updated the manuscript to state this rationale explicitly.

More fundamentally, dichotomizing the dependent variable like this discards data and variation within the data that is then no longer available in the analysis. I suspect this was done to simplify the analysis, but it would be interesting to see results of an analysis where this information was retained, e.g. a multiple linear regression analysis with the (un-dichotomized) dyad-level QMI. Similarly the independent variable is dichotomized by requiring two clusters in the k-means clustering step. There are alternatives, such as logistic regression, where the independent variable can be retained, even if the dependent variable is dichotomized. It would be instructive to see results of an analysis where neither the independent nor the dependent variable have been dichotomized. There may be good reasons to do this, but it should not be done as the first step and the reasons should be provided to the reader.

Thank you for this thoughtful comment. We agree that analyses retaining continuous QMI scores and continuous synchrony measures are informative when the primary goal is prediction or parameter estimation. The aim of the present work, however, is structural characterization and similarity analysis of dyadic interaction dynamics, rather than supervised prediction. Accordingly, we focus on pairwise distance measures between continuous synchrony profiles and their low-dimensional representations to examine relational structure among dyads.

The synchrony features are not used to predict QMI, nor are they discretized to fit a classifier. Instead, each dyad is represented by a continuous, high-dimensional synchrony profile derived from sliding-window zero-lag correlations, which preserves temporal variability throughout the analysis. Clustering was used as an additional structure-discovery step, rather than as a discretization of the independent variable or a substitute for continuous modeling. Specifically, k-means clustering was applied as an unsupervised, exploratory method to identify dominant dynamical regimes in the space, without imposing thresholds or class labels. The choice of k = 2 was aligned with the outcome grouping and further supported by visual inspection, serving to formalize an emergent pattern rather than to force a dichotomy. We have updated the manuscript to state this rationale explicitly.

Motivation for the method

In brief, the method takes simultaneous videorecordings of individuals in a couple and use an automated algorithm to extract AU activation for each AU and each frame of the video. This gives a time series of AU activations that can be matched up for the two individuals in each dyad. These are used to construct a so-called S-matrix of zero-lag correlations between AUs (rows) in a specific time window, and time windows (columns) in a sliding window approach where the window of width w, is moved by some step size along the time series. In a next step pairwise Euclidean distances between vectors that are columns of the S-matrix are calculated and subjugated to multidimensional scaling (MDS) to obtain a two-dimensional representation. The data points in this two-dimensional space are then used as input to a k-means clustering algorithm fixed at producing two clusters from the data. The cluster membership of each couple is then compared to the dichotomized quality of marriage index (QMI) categorizing couples as either stressed or non-stressed.

There are several places along the way, when I read the description of the method summarized above, where I think a little motivation would help to understand the method. I constantly kept wondering why the authors made some very specific choices where there were other alternatives I could think of. I do not ask the authors to consider such alternatives in detail, but merely to provide some reasoning for the steps in the analysis. A figure explaining the method in a conceptual way could go a long way to increase the readability of the manuscript, e.g. along the lines of Figure 1 in Shadeydeh et al. (2021).

Thanks for this great suggestion. We have attached a figure shows the main steps of the work as suggested, together with reasoning for the steps. Briefly, the use of cross-correlations between AUs was motivated by our goal of quantifying facial synchrony within dyads. Applying this analysis in a sliding-window framework allowed us to capture the temporal dynamics of synchrony over the course of the interaction, rather than assuming stationarity. The resulting S-matrix can thus be interpreted as a time-resolved signature of dyadic interaction patterns.

To assess similarity between dyads, we computed distances between these S-matrix profiles, yielding a distance matrix that quantifies how similar or dissimilar different dyads are in terms of their interaction dynamics. Multidimensional scaling (MDS) was then used to obtain a two-dimensional representation of these distances, facilitating visualization and subsequent clustering, as well as comparison with relationship quality measures.

Some of the choices I would like to see explained are:

i) What is the purpose of calculating the pairwise distances between the columns of the S-matrix?

Pairwise distances between S-matrices are computed to quantify how similar or dissimilar dyads are in their overall multivariate, time-varying synchrony patterns, enabling visualization and clustering of interaction dynamics without relying on individual features or predefined labels. We have updated the manuscript to address this point.

ii) why do the authors use MDS to perform dimensional reduction when k-means clustering is fully capable of operating on higher-dimensional data.

Thank you for this insightful question. We can apply k-means to S-matrices, but not directly in their raw matrix form unless we handle the matrix as image or extract the pattern from matrix. We need a representation where each S-matrix becomes a vector or a point in a space where distances are meaningful. MDS is used to obtain a low-dimensional, distance-preserving representation of dyadic synchrony patterns that improves interpretability and robustness, while k-means is applied subsequently for clustering within this embedded space. In sum, the goal of MDS is to see the emerging behavior of dyads in low dimension where we were able to recognize it even by visual inspection.

iii) I also wonder what would happen if a different number of clusters had been found. I understand that the authors are interested in having two clusters to be able to compare to the dichotomized QMI score, but at least a consideration of the number of clusters seems appropriate to include.

We thank the reviewer for this comment. The number of clusters was fixed to two a priori, as the study is theoretically grounded in a binary distinction between stressed and non-stressed states reflected in the QMI outcome. Given the small sample size (27 dyads) and the clear visual separation observed in the low-dimensional representation, our goal was not to estimate an unknown number of clusters but to test whether the data structure aligns with this predefined dichotomy. We added statement showing this rationale in the manuscript.

Description of the method

Apart from the reasoning behind the method, I would also like to see it described a bit more concisely. In several places I had to stop and think about exactly what was meant by a particular phrase or expression. Here are a few examples:

p.8, l.198: "where X_j and Y_j represents the AU vectors of the two individuals within the window w_i." This makes sense, but it is the first time the reader encounters X_j and Y_j and w_i has not been defined. There is w which is the window size, so I think what is really meant is just window i. Here, the authors could help readers a little, by explicitly defining X_j and Y_j by their components which are the values of a particular AU at each of the time stamps in window j. It can be difficult to catch such small things which may confuse the reader when you know yourself what you mean, so I would suggest the authors to get a friendly review by a colleague who is unfamiliar with the method, and ask them to make a note of anything that is not immediately clear.

Thank you for your valuable comment. We have revised the manuscript so that the synchrony equation is introduced explicitly, with all indices and variables defined immediately following the equation.

p.9, l.228 & Eq. 3: "We concatenated each column (AUs) of S-matrix into a single vector v, referring to this as the flattened S-matrix." Here there is inconsistency between the quoted sentence and the notation used in Eq. 3. In the sentence an italic v is used, and in the equation boldface v_i and v_j are used. Also, I do not think it makes sense to say that each columns is concatenated. With what? It is simply that v_i and v_j are columns from the S-matrix, i.e. column vectors. On the other hand, if v is the concatenation of all of these column vectors, then it makes sense to call it the flattened S-matrix, but this does not seem to be used anywhere and only leads to confusion --- at least for me.

Thank you for this comment. We have revised the text and clarified Eq. 3 to remove the inconsistency. We now state explicitly that the entire S-matrix is flattened by concatenating all of its columns into a single vector v. The notation has been made consistent, and v_mand v_nin Eq. 3 now clearly denote the flattened S-matrices of dyads m and n.

Description of preprocessing

The description of the preprocessing is not completely clear to me, so I hope these comments can help the authors improve the description of the preprocessing steps. The discussion of preprocessing is interrelated with the question of data quality and exclusion criteria. In section 3 Data, it is stated that 27 out of approximately 150 couples were selected for analysis due to missing or fragmented data "at the millisecond level." I believe that exluding such a large fraction of the data merits a more detailed description than the rather vague "due to missing or fragmented affective facial expression data at the millisecond level." First of all, I am not convinced that a single datum missing at the millisecond level would be a problem and second, I do not think that the data are even resolved at the millisecond level. Most commercial cameras record at 25, 30, or 60 fps corresponding to a time between frames of 40, 33, and 17 ms, respectively. So even at the highest framerate, the interval between samples is an order of magnitude above the millisecond level. On p.8, l.200-201 the authors say that 1000 samples is equivalent to 40 s, in other words the frame rate is 25 fps. This leaves me wondering what is meant by missing or fragmented data at the milisecond level. Including exact exclusion criteria in the manuscript and in the replication script would be a better way to describe the data exclusion.

Thank you for this helpful clarification. We now provide a detailed description of the preprocessing and exclusion criteria, including: (i) removal of uninformative frames in which all action units were zero, (ii) temporal synchronization of male and female partners by retaining only time points where both had valid data, and (iii) exclusion of dyads with insufficient overlapping synchronized data after preprocessing. These criteria explain why 27 dyads were retained from the original dataset and are now described explicitly in the manuscript and documented in the replication scripts.

In section 4.1 Preprocessing the initial comment that "Missing AU data occurred sparsely" (p.6, l.162) is imprecise. Please provide numbers, e.g, in % (mean and sd over the sample). Briefly thereafter the authors write "Additionally, there were moments when the data was [sic] unavailable for both partners simultaneously within the same dyad." (p. 7, l.164). I do not understand the difference between the two types of missing data. Is it just that sometimes there are data missing for only on partner and sometimes for both? And if so, why is this difference important?

Thank you for this comment. We have revised the manuscript to clarify the distinction and to provide quantitative information. We distinguish between individual-level missingness, where AU data are unavailable for one partner due to insufficient frame quality (e.g., occlusion or tracking failure), and dyad-level missingness, where AU data are unavailable for both partners simultaneously. Across the 27 dyads, individual-level non-informative data averaged 34.1% (SD = 18.3) for male participants and 24.6% (SD = 20.6) for female participants. Dyad-level missingness primarily resulted from concurrent individual-level tracking failures and is important because synchrony measures require valid data from both partners.

I fail to understand exactly why the authors treat the case when all AUs are zero as missing data. Is this a convention in the iMotions data format? And if so, how can this be distinguished from cases where all AUs are actually zero?

We appreciate this opportunity to clarify a technical nuance of the iMotions (AFFDEX) data stream. In automated FER, there is a fundamental distinction between a neutral (or non-emotive) face and a tracking failure. Empirically, the AFFDEX engine almost never produces a true zero value across all affect codes and action units simultaneously. Consequently, a string of absolute zeros across the entire vector is a known signature of a tracking dropout—typically caused by significant head rotation, occlusion (e.g., a hand or hat), or the participant moving out of the camera's frame. Treating these as zeros rather than missing would erroneously signal a state of perfect affective void, which would mathematically distort the synchrony estimates. We have added a brief clarification to the "Data Processing" section to make this distinction explicit.

I do not think Figure 1 is necessary to explain the simple concept of dropping rows with missing data. If kept, it should be converted into a table instead of a figure.

Thank you for this suggestion. We agree that Figure 1 is not necessary to explain this preprocessing step. Accordingly, Figure 1 has been removed from the revised manuscript.

The process of synchronizing the time stamps (p. 7, l.175) is not explained at all. Please provide an explanation. Perhaps because I do not understand the issue of synchronizing time stamps, I also fail to understand the decision "to use only the first minute of available data for each dyad." (p. 7, l. 180). This criterion along with the exclusion of most of the couples mean that only around 1.8% of the original dataset is used for the analysis. If the method does not handle "issues ... commmon in video analysis" (p. 7, l.165) it seems to be a major problem for the application of the method that the authors propose. I therefore urge the authors to think about how missing data can be handled in a more robust way. If the issue with dropping NA rows and then synchronizing the two individual time series in each couple is that the time series increasingly drift apart (or out-of-sync) then perhaps it is possible to take several 1-min epochs from the 10-minute conversations and align the two time series at the beginning of each epoch. If this is possible it would increase the data used in the analysis by up to a factor of 10.

Thank you for this comment. We have revised Section 4.1 to clarify the timestamp synchronization procedure and the rationale for using only the first minute of data. AU time series were extracted independently for each partner, and intermittent tracking failures resulted in misaligned timestamps and unequal numbers of valid frames. Synchronization was performed by aligning timestamps and retaining only time points with valid AU data for both partners, as required for zero-lag synchrony analysis.

The use of a one-minute segment reflects a limitation of the dataset rather than the proposed method. Most dyads provided approximately 60 s of continuous synchronized data after preprocessing, where the total synchronized duration across dyads had a mean of 113.3 s (SD = 65.9 s), making it difficult to reliably extract multiple independent one-minute epochs. We therefore selected the first continuous one-minute segment to ensure consistency across dyads. We agree that epoch-based re-synchronization is a promising extension and have now identified it as an important direction for future work.

Relevant baseline comparison

----------------------------

I think it is relevant to ask what the benefit is of extra detail, such as moment-to-moment synchrony as opposed to overall synchrony. In this case, where the dependent variable is a single measure (dichotomized QMI score) an approach using only average synchrony over the whole one-minute interval might be a more reasonable comparison. It would be interesting to know how such an approach (perhaps based on cross recurrence quantification analysis) compares to the moment-to-moment approach and to the current baseline approach using traditional statistical summary measures.

Thank you for this insightful comment. While moment-to-moment synchrony provides a richer characterization of interaction dynamics, we agree that comparisons with aggregate synchrony approaches (e.g., average synchrony over an interval or CRQA-based measures) are valuable. Given the limited continuous duration available in the current dataset, such comparisons could not be explored exhaustively and are therefore identified as important directions for future work

Including other approaches

--------------------------

This ties in with my previous comment about the relevant baseline comparison, viz. that other approaches have not been mentioned under related work. Specifically, the approach taken by the authors of using zero-lag cross-correlation has the implication that complementarity rather than similarity is not picked up by the method (see e.g. Dale et al. 2013). Others have analyzed facial expression dynamics in a way that takes such effects into account, e.g. Postma and Postma-Nilsenová (2016) and Marx et al. (2025). These are just two examples of papers that take such an approach using two different methods which are able to capture complementarity in addition to zero-lag synchrony. This type of literature seems to be completely ignored by the authors, and including and relating to this literature, e.g. in section 2.3 Characterization of Facial Synchrony would enable the reader to understand how the authors' approach differ.

Thank you for this helpful comment. We have revised Section 2.3 (Characterization of Facial Synchrony) to explicitly acknowledge and relate our work to prior approaches that model lagged and complementary coupling (e.g., Dale et al., 2013; Postma & Postma-Nilsenová, 2016; Marx et al., 2025). We clarify that the present study focuses on moment-to-moment zero-lag synchrony as a system-level baseline, and we discuss how this choice differs conceptually and methodologically from lag-based approaches. Despite this restriction, we show that time-localized zero-lag synchrony is sufficient to reveal clear separation between dyads in our dataset.

Figures are not publication quality

-----------------------------------

The quality of the figures in the PDF is low resolution, so I had to download each figure separately in order to be able to clearly see the details. This may be a problem with the journal's production system or it could be because the files are in a bitmap format (TIFF) rather than a vector format such as PDF.

Resolution issues aside, the figures all need some more work to make them publication quality. In many of the figures the text is so small that it is very hard to read, so the font size should be increased.

Rather than providing detailed feedback on each figure, I will do this for one figure and leave it to the authors to take similar actions for the remaining figures.

Below is detailed feedback on Figure 4.

Organize the sub-plots by dichotomized QMI score (0 and 1) in two separate panels.

Now the title (0 or 1) can be removed to give more space to the data.

Remove all the common horizontal and vertical axes, except at the bottom and at the far left side. This frees up even more space and avoids redundancy.

Add axes labels on the reamining axes and increase the font size.

Consider a portrait layout instead of landscape to give a better aspect ratio for the individual sub-plots.

Add a common color bar to indicate the scale (at top or bottom)

Decrease the white space between the plots to give more space to the data.

Thank you for this helpful suggestion. We have updated the figures as suggested, considering the journal specifications for figures submission.

## Minor comments

Since there are so many major comments that need to be addressed, it does not make sense to address all of the minor comments, so I will limit myself to a few (mostly recurring) issues.

* Equations: In many cases the equations are not inserted immediately after they are first referenced. This seems unconventional and makes it harder to read the manuscript (not much, but mainly it just flouts a convention).

Thank you for noting this. We have revised the manuscript to ensure that all equations are now placed immediately after their first reference in the text, improving readability and conforming to standard formatting conventions.

* Tables: Table 1 seems unnecessary, since the contents is well-known to any knowledgable reader. Tables 2 and 3 have horizontal rules between rows that should not be there.

Thank you for the comment. We have removed Table 1 from the manuscript, as its contents are well known to the target audience. In addition, the unnecessary horizontal rules between rows in Tables 2 and 3 have been removed to conform to standard table formatting guidelines.

* Number of digits: In some cases too many digits are provided, considering precision and sensitivity of the analysis. E.g., on p.11, l.273 and p.13, l.320 the baseline silhouette score is given as 0.4622. One or two significant digits would be sufficient here.

Thank you for the suggestion. We have reduced the number of reported digits and rounded values (e.g., silhouette scores) to an appropriate level of precision throughout the manuscript.

* Citing sources that are not open: I tried to access the link to the iMotions resource on facial expression analysis (reference 26), but was required to provide my personal information in order to obtain a copy. Consider if it is possible to cite an openly available and perhaps even peer-reviewed source instead.

We thank the reviewer for raising this important point regarding accessibility. We agree that citing openly available and peer-reviewed sources is valuable for transparency and reproducibility. We believe that the iMotions guidebook remains a useful reference, particularly for readers who have access to the software or who wish to understand its technical capabilities and implementation details; therefore, we have retained this citation.

To address the reviewer’s concern, we have additionally included an openly accessible, peer-reviewed study that explicitly documents the frame-by-frame facial expression analysis capability of the Affectiva engine integrated within iMotions. Specifically, we now cite:

Kulke L, Feyerabend D, Schacht A. A comparison of the Affectiva iMotions Facial Expression Analysis Software with EMG for identifying facial expressions of emotion. Frontiers in psychology. 2020;11:329.

References:

Bibby, E. S., McKinnon, A. M., Shaw, M., & Mattson, R. E. (2023). Accurately detecting happy facial expressions associates with perceptions of flirtatiousness. Journal of Social and Personal Relationships, 40(4), 1288-1315.

Coan, J. A., & Sbarra, D. A. (2015). Social baseline theory: The social regulation of risk and effort. Current opinion in psychology, 1, 87-91.

Karney, B. R., & Bradbury, T. N. (1995). The longitudinal course of marital quality and stability: A review of theory, methods, and research. Psychological bulletin, 118(1), 3.

Woods, S. B., Priest, J. B., & Denton, W. H. (2013). A preliminary investigation of a telephone administration of the Quality of Marriage Index. Journal of marital and family therapy, 39(2), 261-267.

Attachment Submitted filename: Response to Reviewers.docx

# Peer review: Decision Letter 1

**DOI:** 10.1371/journal.pone.0356071.r003

**Authors:** Sebastian Wallot

26 Feb 2026

Dear Dr. Fora,

Thank you for submitting your manuscript to PLOS ONE. After careful consideration, we feel that it has merit but does not fully meet PLOS ONE’s publication criteria as it currently stands. Therefore, we invite you to submit a revised version of the manuscript that addresses the points raised during the review process.

Please submit your revised manuscript by Apr 12 2026 11:59PM. If you will need more time than this to complete your revisions, please reply to this message or contact the journal office at plosone@plos.org. When you're ready to submit your revision, log on to https://www.editorialmanager.com/pone/ and select the 'Submissions Needing Revision' folder to locate your manuscript file.

- A letter that responds to each point raised by the academic editor and reviewer(s). You should upload this letter as a separate file labeled 'Response to Reviewers'.
- A marked-up copy of your manuscript that highlights changes made to the original version. You should upload this as a separate file labeled 'Revised Manuscript with Track Changes'.
- An unmarked version of your revised paper without tracked changes. You should upload this as a separate file labeled 'Manuscript'.

If applicable, we recommend that you deposit your laboratory protocols in protocols.io to enhance the reproducibility of your results. Protocols.io assigns your protocol its own identifier (DOI) so that it can be cited independently in the future. For instructions see: https://journals.plos.org/plosone/s/submission-guidelines#loc-laboratory-protocols. Additionally, PLOS ONE offers an option for publishing peer-reviewed Lab Protocol articles, which describe protocols hosted on protocols.io. Read more information on sharing protocols at https://plos.org/protocols?utm_medium=editorial-email&utm_source=authorletters&utm_campaign=protocols.

We look forward to receiving your revised manuscript.

Kind regards,

Sebastian Wallot, Ph.D

Academic Editor

PLOS One

Journal Requirements:

If the reviewer comments include a recommendation to cite specific previously published works, please review and evaluate these publications to determine whether they are relevant and should be cited. There is no requirement to cite these works unless the editor has indicated otherwise.

Please review your reference list to ensure that it is complete and correct. If you have cited papers that have been retracted, please include the rationale for doing so in the manuscript text, or remove these references and replace them with relevant current references. Any changes to the reference list should be mentioned in the rebuttal letter that accompanies your revised manuscript. If you need to cite a retracted article, indicate the article’s retracted status in the References list and also include a citation and full reference for the retraction notice.

[Note: HTML markup is below. Please do not edit.]

Reviewers' comments:

Reviewer's Responses to Questions

**Comments to the Author**

Reviewer #1: (No Response)

Reviewer #2: (No Response)

**********

2. Is the manuscript technically sound, and do the data support the conclusions?

Reviewer #1: Yes

Reviewer #2: Yes

**********

3. Has the statistical analysis been performed appropriately and rigorously?

Reviewer #1: Yes

Reviewer #2: Yes

**********

4. Have the authors made all data underlying the findings in their manuscript fully available?

The PLOS Data policy

Reviewer #1: Yes

Reviewer #2: Yes

**********

5. Is the manuscript presented in an intelligible fashion and written in standard English?

Reviewer #1: Yes

Reviewer #2: No

**********

Reviewer #1: 1. The manuscript indicates that the full dataset included 150 couples, of which only 27 were retained for analysis, because “for some participants AU data were entirely missing due to unavailable video, resulting in incomplete dyads.” It is unclear whether videos were not collected for these dyads, or whether videos were collected but could not be processed due to technical failure. The authors should provide a transparent accounting of the exclusion process (e.g. how much missing data was too much and how many dyads were excluded for this reason). Providing this information would allow readers to clearly understand how the final analytic sample was derived and to evaluate the robustness and generalizability of the findings. Given the substantial reduction from the original dataset to the final sample, explicit reporting of exclusion criteria and counts at each stage is important to ensure transparency and to preempt potential concerns about sample composition.

2. The shared dataset appears to include all participants, without documentation of which dyads were retained or excluded in the analyses. Without this information, it is not possible to retrace the reported results from the shared data. Authors should provide an explicit index of included dyads and a reproducible filtering script. In addition, the dataset would benefit from accompanying documentation (e.g., a README file) that clearly explains the structure of the data, and the meaning of each variable.

3. In the revised manuscript, the description of how missing data were handled in the datasets used for analysis appears to have been removed. Readers need to know whether missing frames were discarded, interpolated, imputed, or handled in some other way prior to computing cross-correlations and constructing the S-matrix.

4. The manuscript introduces the term “similarity-based synchrony” to refer to zero-lag coupling, while later also using the term “concurrent synchrony.” Although the underlying construct is clear, the terminology is nonstandard and potentially confusing. Adopting concurrent synchrony as a single, standard term would improve conceptual clarity and alignment with the existing literature.

5. Figures 1, 4, 5, and 9 in the current version still appear low in resolution. In addition, the dyad identifiers in Figure 2 are visually dense, and labels in Figure 5 should be capitalized (Male, Female).

6. Typos (lines according to the tracked changes document):

– Line 213: the reference should appear before the full stop at the end of the sentence.

– Line 259: missing space before the word “Data.”

- Multiple adjacent citations are listed as separate bracketed references (“[25] [26] [27]”) rather than consolidated ranges (“[25–27]”).

Reviewer #2: (No Response)

**********

what does this mean?). If published, this will include your full peer review and any attached files.

If you choose “no”, your identity will remain anonymous but your review may still be made public.

**Do you want your identity to be public for this peer review?**  For information about this choice, including consent withdrawal, please see our Privacy Policy

Reviewer #1: **Yes:**  Alon Burns

Reviewer #2: **Yes:**  Dan Mønster

**********

[NOTE: If reviewer comments were submitted as an attachment file, they will be attached to this email and accessible via the submission site. Please log into your account, locate the manuscript record, and check for the action link "View Attachments". If this link does not appear, there are no attachment files.]

To ensure your figures meet our technical requirements, please review our figure guidelines: https://journals.plos.org/plosone/s/figures

You may also use PLOS’s free figure tool, NAAS, to help you prepare publication quality figures: https://journals.plos.org/plosone/s/figures#loc-tools-for-figure-preparation.

NAAS will assess whether your figures meet our technical requirements by comparing each figure against our figure specifications.

Attachment Submitted filename: review.pdf

# Author comment: Author response to Decision Letter 2

**DOI:** 10.1371/journal.pone.0356071.r004

23 Apr 2026

We thank the Reviewers for their careful reading of our manuscript and for their constructive and insightful comments. We have considered all comments and revised the manuscript accordingly.

For Reviewer 1, we provided point-by-point responses following the order of the comments. For Reviewer 2, we have organized our responses under the main headings, maintaining the same order and titles as presented in the review to facilitate clarity and ease of reference.

Response to Reviewer 1

Point #1

Thank you for this important comment. We have revised the manuscript to improve transparency in the exclusion process, and provide a clear summary of the number of dyads excluded at each stage, so that the derivation of the final sample is transparent.

Point #2

Thank you for this important comment. Figure 2 in the manuscript illustrates the dyads included in the analysis. Detailed information is provided in the code file Part1_Check_Data.m. Additionally, the README file has been updated to include more comprehensive definitions of the variables.

Point #3

Thank you for pointing this out, as it is an important aspect of the work that warrants clear explanation. We would like to clarify that the description of how missing data were handled was not removed, but rather revised and clarified in the previous version of the manuscript. As stated in Section 3.3, missing frames were excluded prior to the computation of cross-correlations and the construction of the S-matrix.

Point #4

Thank you for the suggestion. We have standardized the terminology by using concurrent synchrony throughout and removed “similarity-based synchrony” to improve clarity and alignment with the literature.

Point #5

Thank you for pointing this out. We replaced Figures 1, 4, 5, and 9 (in the previous revision) with higher-resolution versions to improve clarity. We have carefully worked on improving the image quality; however, it is possible that the remaining issues are related to the PDF generation process. If the figures are still not sufficiently clear, we would greatly appreciate your guidance, and we will address it immediately. We apologize for this issue.

In addition, we revised Figure 2 to reduce visual density in the dyad identifiers for better readability, and updated Figure 5 to capitalize the labels (Male, Female).

Point #6

Thank you for identifying these typos. Some of them were introduced during the track changes process, and all have now been addressed.

Response to Reviewer 2

Major Comments

QMI Threshold

Thank you for this thoughtful and important comment. We appreciate the opportunity to clarify the rationale and process underlying the selection of the QMI threshold.

First, we would like to clarify that the threshold of 80 was not chosen with the explicit goal of forcing an equal split of the sample. Rather, it emerged from a combination of data-driven inspection and theoretical considerations at the dyadic level.

From a data-driven perspective, we observed that the MDS representation of pairwise distances derived from the S-matrices revealed a natural separation into two clusters. When overlaid with summed QMI scores, dyads in one region were generally associated with lower scores, while those in the opposing region exhibited higher scores. This pattern suggested the presence of two qualitatively distinct groupings. In parallel, examination of the empirical distribution showed that the median summed QMI score was 79 in the analyzed subsample and 83 in the full sample of 150 couples. Therefore, a threshold near 80 aligns with the central tendency of both datasets, rather than being arbitrarily imposed.

Importantly, our intention was not to define a universal or transferable cutoff. We adopt a sample-specific threshold because the analysis is grounded in pairwise relational structure and unsupervised clustering, where labels are used post hoc for interpretation rather than imposed a priori. So the use of a sample-specific threshold is consistent with our analysis framework, where pairwise distance representations and unsupervised clustering define the underlying structure, and QMI-based grouping is applied post hoc to interpret this structure rather than to determine it.

We fully agree with the reviewer’s concern that reporting a threshold without context may lead to inappropriate reuse. For this reason, we have revised the manuscript to more explicitly describe the process used to arrive at this value. Specifically, the threshold reflects:

alignment with the empirical distribution, and

consistency with the structure observed in the unsupervised clustering space.

Finally, we emphasize that this threshold is sample-specific and was introduced to facilitate the sensitivity of the clustering analysis at the dyadic level, rather than to serve as a clinical or generalizable benchmark. We have now made this distinction more explicit in the revised manuscript to ensure that readers can understand and potentially adapt the methodology, rather than replicate the specific numeric value.

We thank the reviewer again for highlighting this point, as it has helped us improve the clarity and reproducibility of our work.

Base line Comparison:

We thank the reviewer for this important comment regarding the choice of baseline and feature comparison.

In this work, we adopted statistical feature extraction as a baseline, as it is a widely used approach in the literature. From a machine learning perspective, this corresponds to constructing feature representations based on summary statistics. We then compared the pairwise distance measurement based on these statistical features with the proposed multidimensional synchrony-based method.

A key consideration in this comparison is feature dimensionality. For the statistical approach, extracting n statistical measures across 20 action units results in a feature space of size 20×n. Introducing windowing further increases this dimensionality, potentially leading to high-dimensional representations relative to the available data. In contrast, the zero-lag cross-correlation (synchrony-based) approach provides a more compact representation. Specifically, it produces one value per action unit per window, resulting in a feature space of 20×1 per window.

We acknowledge that one possible extension would be to apply window-based statistical analysis. However, this would significantly increase dimensionality and may not be suitable given the limited data duration (~60 seconds). Conversely, we evaluated a no-window version of the synchrony-based approach (as suggested by the reviewer), and obtained results consistent with the windowed analysis. The no-window synchrony-based analysis outperforms the statistical features but shows lower performance than the windowed cross-correlation approach.

The synchrony-based features demonstrated improved clustering performance compared to statistical features. This indicates that global, pairwise synchrony measurements capture meaningful structure in the data, even with fewer features.

Clustering quality was primarily assessed using the Silhouette score, which reflects both intra-cluster compactness and inter-cluster separation. To complement this, we reported the Euclidean distance between cluster centroids as an interpretable geometric measure of separation. Figure 6 was added to show the impact of the used configuration on the grouping pattern.

Minor Comments

Add explanation for exclusion earlier

Thank you for your comment. We have revised the sentence by adding the word “only” for clarity. In addition, we included a reference to the section where the data selection process is described in detail.

Figure 2

Thank you for this helpful suggestion. We have revised Figure 2 by sorting the dyads according to the length of the available synchronized data. This modification improves the readability of the figure and makes the comparison across dyads more intuitive.

K-means clustering results

Thank you for these insightful suggestions. We have further investigated both points to better understand the observed pattern.

First, regarding the use of MDS, we extended the analysis to a three-dimensional representation. The results showed a pattern consistent with the 2D projection. This suggests that the observed overlap is not due to the dimensionality reduction but rather reflects the underlying structure. This point has now been addressed in the revised manuscript.

Second, concerning the QMI threshold, we found that the misclassified cases are not all located near the threshold. This indicates that the misclassification cannot be explained solely by the dichotomization of QMI.

Thanks for the suggestion to include the QMI figure. We have added this figure in the current version, illustrating three different cases to show the impact of varying window sizes and to enable comparison with the no-window baseline.

Report window sizes

We thank the reviewer for this important clarification.

Our intention was to convey that the confusion matrices obtained across different window sizes were highly similar, indicating stable classification behavior. However, we agree that presenting a single confusion matrix does not demonstrate this consistency and that our original wording was unclear. We changed the wording in the manuscript to show that better.

In addition, we have updated all relevant figure captions to clearly specify the window size used.

Practical implications and limitations

We thank the reviewer for the thoughtful and constructive comments. Regarding the concern about clinical applicability, we agree that the current results do not provide sufficient evidence to support strong clinical claims. Our intention was not to suggest immediate clinical deployment, but rather to highlight a potential long-term direction. We have revised the manuscript to clarify that the current findings should be interpreted as proof-of-concept, demonstrating sensitivity of the proposed representation to dyadic interaction patterns, rather than as a clinically validated tool.

With respect to the suggestion of using continuous measures instead of dichotomization, we acknowledge this as an important limitation and have now explicitly stated it in the revised manuscript. However, given the small sample size (n = 27), our primary goal was to investigate whether the proposed representation can differentiate between qualitatively distinct dyadic states, rather than to model fine-grained variations in relationship quality.

In other words, the focus is on identifying whether meaningful structure exists in the interaction dynamics that aligns, even partially, with self-reported outcomes. Modeling QMI as a continuous variable would require a larger dataset to ensure robustness and avoid overfitting, which is beyond the scope of the current work.

Regarding the confusion matrix and the observed false positive rate for non-stressed couples, we agree that performance is not uniform across groups. The purpose of the confusion matrix in this study is not to frame the approach as a classification model, but rather to provide an external evaluation of the clustering results and a general way of assessing how dyads are categorized. It serves to illustrate the agreement between the cluster assignments and the true labels. Although both the baseline and the synchrony-based methods exhibit similar false positive rates, the distribution of dyads in the space is more clearly structured when using the synchrony-based representation.

Other minor issues

Thank you for pointing this out. We have corrected the notation to ensure that a proper mathematical minus sign is used instead of a hyphen in the sentence following Eq. 6.

Figure 5:

Thank you for the clarification. We have revised the caption of Figure 5 to replace “x-axis” and “y-axis” with “horizontal axis” and “vertical axis,” ensuring more precise and appropriate terminology.

Thank you for the observation. We have removed the references to “(A)” and “(B)” from the caption and revised it to describe the tree icons representing the two classes, ensuring alignment with the figure.

Attachment Submitted filename: Response_to_reviewers_auresp_2.docx

# Peer review: Decision Letter 2

**DOI:** 10.1371/journal.pone.0356071.r005

**Authors:** Sebastian Wallot

3 Jun 2026

Dear Dr. Fora,

Please submit your revised manuscript by Jul 18 2026 11:59PM. If you will need more time than this to complete your revisions, please reply to this message or contact the journal office at plosone@plos.org. When you're ready to submit your revision, log on to https://www.editorialmanager.com/pone/ and select the 'Submissions Needing Revision' folder to locate your manuscript file.

- A letter that responds to each point raised by the academic editor and reviewer(s). You should upload this letter as a separate file labeled 'Response to Reviewers'.
- A marked-up copy of your manuscript that highlights changes made to the original version. You should upload this as a separate file labeled 'Revised Manuscript with Track Changes'.
- An unmarked version of your revised paper without tracked changes. You should upload this as a separate file labeled 'Manuscript'.

If you would like to make changes to your financial disclosure, please include your updated statement in your cover letter. Guidelines for resubmitting your figure files are available below the reviewer comments at the end of this letter.

If applicable, we recommend that you deposit your laboratory protocols in protocols.io to enhance the reproducibility of your results. Protocols.io assigns your protocol its own identifier (DOI) so that it can be cited independently in the future. For instructions see: https://journals.plos.org/plosone/s/submission-guidelines#loc-laboratory-protocols. Additionally, PLOS ONE offers an option for publishing peer-reviewed Lab Protocol articles, which describe protocols hosted on protocols.io. Read more information on sharing protocols at https://plos.org/protocols?utm_medium=editorial-email&utm_source=authorletters&utm_campaign=protocols.

As the corresponding author, your ORCID iD is verified in the submission system and will appear in the published article. PLOS supports the use of ORCID, and we encourage all coauthors to register for an ORCID iD and use it as well. Please encourage your coauthors to verify their ORCID iD within the submission system before final acceptance, as unverified ORCID iDs will not appear in the published article. *Only*  the individual author can complete the verification step; PLOS staff *cannot*  verify ORCID iDs on behalf of authors.

We look forward to receiving your revised manuscript.

Kind regards,

Sebastian Wallot, Ph.D

Academic Editor

PLOS One

Journal Requirements:

If the reviewer comments include a recommendation to cite specific previously published works, please review and evaluate these publications to determine whether they are relevant and should be cited. There is no requirement to cite these works unless the editor has indicated otherwise.

Please review your reference list to ensure that it is complete and correct. If you have cited papers that have been retracted, please include the rationale for doing so in the manuscript text, or remove these references and replace them with relevant current references. Any changes to the reference list should be mentioned in the rebuttal letter that accompanies your revised manuscript. If you need to cite a retracted article, indicate the article’s retracted status in the References list and also include a citation and full reference for the retraction notice.

[Note: HTML markup is below. Please do not edit.]

Reviewers' comments:

Reviewer's Responses to Questions

**Comments to the Author**

Reviewer #1: (No Response)

Reviewer #2: (No Response)

**********

2. Is the manuscript technically sound, and do the data support the conclusions?

Reviewer #1: Partly

Reviewer #2: Yes

**********

3. Has the statistical analysis been performed appropriately and rigorously?

Reviewer #1: No

Reviewer #2: Yes

**********

4. Have the authors made all data underlying the findings in their manuscript fully available?

The PLOS Data policy

Reviewer #1: Yes

Reviewer #2: Yes

**********

5. Is the manuscript presented in an intelligible fashion and written in standard English?

Reviewer #1: Yes

Reviewer #2: Yes

**********

Reviewer #1: While exclusions due to technical limitations are expected in this type of data, the magnitude of this reduction is substantial and requires clear justification. Despite the revisions, the exclusion criteria remain insufficiently specified. Terms such as “insufficient data” and “substantial portions of unusable data” are used, but no explicit quantitative thresholds are provided. At this stage, it is essential that the authors clearly define how much missing or unusable data led to exclusion (e.g., proportion of missing frames, minimum duration of valid data). Without such thresholds, the exclusion process cannot be evaluated or reproduced. In addition, the distinction between “insufficient data” and “substantial portions of unusable data” remains unclear. As written, these appear to reflect overlapping concepts, and it is not possible to determine how decisions were made at each stage of the pipeline.

More importantly, the manuscript does not provide sufficient justification for the extent of data reduction. It remains unclear whether the exclusion process necessarily leads to a final sample of 27 dyads, or whether a larger portion of the dataset could have been retained under clearly defined criteria. Given the size of the original dataset, this point requires direct empirical support. The authors should evaluate whether alternative, less restrictive inclusion strategies (e.g., segment-level inclusion or different missing-data thresholds) would increase the usable sample size. If not, this should be demonstrated and explained fully to provide clearer justification for the exclusion pipeline.

Reviewer #2: Review of manuscript PONE-D-25-41087R2

I thank the authors for addressing all the issues raised by myself and another reviewer. I think the changes has improved the manuscript, and I only have two rather small suggestions that can easily be addressed. I look forward to seeing the finished paper.

1. In their response letter the authors write that "we evaluated a no-window version of the synchrony-based approach (as suggested by the reviewer), and

obtained results consistent with the windowed analysis." This suggests that an aggregated approach is almost as good as the windowed approach, but in the abstract they highlight a "sliding-window framework that computes moment-to-moment facial synchrony." It would seem that the sliding window approach is not so crucial, which would also be surprising, given a window size that is 2/3 of the entire time series. So, in their description in the abstract of "moment-to-moment synchrony" I suggest the authors tone down the emphasis on "evolving synchrony state of each dyad over time" and focus on the synchrony. It seems to be the synchrony rather than the windowed analysis that does the heavy lifting vis-a-vis the results, and it would be a more honest description if the abstract reflected this. I like the new paragraphs discussing this is the Results and Discussion section, so there is not need to change the manuscript itself, just the abstract.

2. In a newly added paragraph about the exclusion of data the authors write: "This initial exclusion did not account for additional quality issues, such as lack of synchronization" and "41 were further excluded due to either substantial portions of unusable data or poor synchronization between male and female participants." This is very confusing to me, since synchronization could be understood to refer to the synchrony measure. But I am almost certain the authors mean something else here, and this has to be clarified.

And finally, just as a comment, I am happy to see that the authors have now described the QMI threshold selection in a new section. This greatly improves the manuscript.

**********

what does this mean?). If published, this will include your full peer review and any attached files.

If you choose “no”, your identity will remain anonymous but your review may still be made public.

**Do you want your identity to be public for this peer review?**  For information about this choice, including consent withdrawal, please see our Privacy Policy

Reviewer #1: No

Reviewer #2: **Yes:**  Dan Mønster

**********

[NOTE: If reviewer comments were submitted as an attachment file, they will be attached to this email and accessible via the submission site. Please log into your account, locate the manuscript record, and check for the action link "View Attachments". If this link does not appear, there are no attachment files.]

To ensure your figures meet our technical requirements, please review our figure guidelines: https://journals.plos.org/plosone/s/figures

You may also use PLOS’s free figure tool, NAAS, to help you prepare publication quality figures: https://journals.plos.org/plosone/s/figures#loc-tools-for-figure-preparation.

NAAS will assess whether your figures meet our technical requirements by comparing each figure against our figure specifications.

# Author comment: Author response to Decision Letter 3

**DOI:** 10.1371/journal.pone.0356071.r006

22 Jun 2026

We thank the reviewers for their valuable comments. Below, we provide point-by-point responses to each comment. Reviewer comments are presented first, followed by our responses (highlighted in red for illustration purposes).

Reviewer #1: While exclusions due to technical limitations are expected in this type of data, the magnitude of this reduction is substantial and requires clear justification. Despite the revisions, the exclusion criteria remain insufficiently specified. Terms such as “insufficient data” and “substantial portions of unusable data” are used, but no explicit quantitative thresholds are provided. At this stage, it is essential that the authors clearly define how much missing or unusable data led to exclusion (e.g., proportion of missing frames, minimum duration of valid data). Without such thresholds, the exclusion process cannot be evaluated or reproduced. In addition, the distinction between “insufficient data” and “substantial portions of unusable data” remains unclear. As written, these appear to reflect overlapping concepts, and it is not possible to determine how decisions were made at each stage of the pipeline.

More importantly, the manuscript does not provide sufficient justification for the extent of data reduction. It remains unclear whether the exclusion process necessarily leads to a final sample of 27 dyads, or whether a larger portion of the dataset could have been retained under clearly defined criteria. Given the size of the original dataset, this point requires direct empirical support. The authors should evaluate whether alternative, less restrictive inclusion strategies (e.g., segment-level inclusion or different missing-data thresholds) would increase the usable sample size. If not, this should be demonstrated and explained fully to provide clearer justification for the exclusion pipeline.

We thank the reviewer for this important comment. We agree that the reduction from the original dataset to the final sample of 27 dyads requires clear justification. The dataset used in this study is a pre-existing dataset collected at our institution and was not originally optimized for the purpose of automated facial synchrony analysis. Rather, this present study extends our prior work linking discrete iMotions-detected facial affect events with relationship quality by applying automated facial expression analysis and synchrony measures to investigate dyadic relationship dynamics. To our knowledge, open-source datasets containing dyadic facial-expression recordings from married couple interaction together with relationship quality labels are largely unavailable, likely in part because facial video constitutes sensitive personal data. Given the scarcity of suitable public datasets and our prior work using this dataset, we believe it was reasonable to utilize this dataset for the present synchrony work.

The large number of exclusions primarily reflects limitations in the quality and availability of the facial recordings rather than the application of highly restrictive selection criteria. After processing the videos with iMotions/AFFDEX, many recordings contained substantial periods in which facial landmarks could not be reliably tracked, resulting in frames with all AU values equal to zero (e.g., participants leaning out of frame, turning away from the camera, or briefly occluding their faces with their hands). We treated these frames as non-informative and excluded them from analysis. Although missing-data approaches such as interpolation could be considered, the amount of missing data in many recordings was substantial. Because our goal was to quantify facial synchrony using genuine observed facial behavior, we chose a conservative approach and restricted analyses to valid tracked frames rather than generating synthetic facial-expression values through interpolation.

The primary inclusion criterion was the availability of overlapping valid facial-expression data from both partners within a dyad. We examined the duration of valid overlapping recordings and observed that most retained dyads contained approximately one minute of valid data. Requiring substantially longer durations would have further reduced the sample size, whereas allowing much shorter durations would have increased the sample size at the expense of data quality. Therefore, rather than maximizing the number of retained dyads, we prioritized the availability of sufficiently long periods of valid facial-expression recordings from both partners, balancing sample size against data quality.

To clarify our terminology, "insufficient data" refers to cases in which the original recordings were unavailable, heavily occluded, or contained extensive periods of tracking failure, resulting in little or no usable facial-expression information. In contrast, "substantial data loss" refers to dyads that, after removal of non-informative frames, contained less than the minimum duration threshold required for analysis. We selected a threshold of 60 seconds because it represented a reasonable compromise between retaining an adequate sample size and ensuring sufficient data availability for reliable synchrony estimation. We have revised the manuscript to clarify these distinctions, differentiate between missing data and insufficient overlapping valid recordings, and provide a more transparent description of the exclusion process and its rationale.

Reviewer 2

1. In their response letter the authors write that "we evaluated a no-window version of the synchrony-based approach (as suggested by the reviewer), and

obtained results consistent with the windowed analysis." This suggests that an aggregated approach is almost as good as the windowed approach, but in the abstract they highlight a "sliding-window framework that computes moment-to-moment facial synchrony." It would seem that the sliding window approach is not so crucial, which would also be surprising, given a window size that is 2/3 of the entire time series. So, in their description in the abstract of "moment-to-moment synchrony" I suggest the authors tone down the emphasis on "evolving synchrony state of each dyad over time" and focus on the synchrony. It seems to be the synchrony rather than the windowed analysis that does the heavy lifting vis-a-vis the results, and it would be a more honest description if the abstract reflected this. I like the new paragraphs discussing this is the Results and Discussion section, so there is not need to change the manuscript itself, just the abstract.

We thank the reviewer for this thoughtful observation. In light of this comment, we revised the abstract to reduce emphasis on moment-to-moment dynamics and instead focus on the synchrony representation and its association with marital relationship quality.

2. In a newly added paragraph about the exclusion of data the authors write: "This initial exclusion did not account for additional quality issues, such as lack of synchronization" and "41 were further excluded due to either substantial portions of unusable data or poor synchronization between male and female participants." This is very confusing to me, since synchronization could be understood to refer to the synchrony measure. But I am almost certain the authors mean something else here, and this has to be clarified.

We thank the reviewer for highlighting this ambiguity. We agree that the term synchronization could be misinterpreted as referring to the facial synchrony measure analyzed in this study. In this context, however, synchronization simply refers to the temporal alignment of the recordings from the male and female participants within a dyad (aka whether two datapoints from both participants are both available at the same timestamp), rather than the behavioral synchrony computed from facial action units. To avoid confusion, we have replaced the term synchronization with temporal alignment in the manuscript.

Attachment Submitted filename: Response_to_Reviewers_auresp_3.docx

# Peer review: Decision Letter 3

**DOI:** 10.1371/journal.pone.0356071.r007

**Authors:** Sebastian Wallot

30 Jul 2026

Characterizing Multidimensional Facial Synchrony for Relationship Quality Detection

PONE-D-25-41087R3

Dear Dr. Fora,

We’re pleased to inform you that your manuscript has been judged scientifically suitable for publication and will be formally accepted for publication once it meets all outstanding technical requirements.

Within one week, you’ll receive an e-mail detailing the required amendments. When these have been addressed, you’ll receive a formal acceptance letter and your manuscript will be scheduled for publication.

An invoice will be generated when your article is formally accepted. Please note, if your institution has a publishing partnership with PLOS and your article meets the relevant criteria, all or part of your publication costs will be covered. Please make sure your user information is up-to-date by logging into Editorial Manager at Editorial Manager® and clicking the ‘Update My Information' link at the top of the page. For questions related to billing, please contact billing support.

If your institution or institutions have a press office, please notify them about your upcoming paper to help maximize its impact. If they’ll be preparing press materials, please inform our press team as soon as possible -- no later than 48 hours after receiving the formal acceptance. Your manuscript will remain under strict press embargo until 2 pm Eastern Time on the date of publication. For more information, please contact onepress@plos.org.

Kind regards,

Sebastian Wallot, Ph.D

Academic Editor

PLOS One

Additional Editor Comments (optional):

Reviewers' comments:

Reviewer's Responses to Questions

**Comments to the Author**

Reviewer #1: All comments have been addressed

**********

2. Is the manuscript technically sound, and do the data support the conclusions?

Reviewer #1: Yes

**********

3. Has the statistical analysis been performed appropriately and rigorously?

Reviewer #1: Yes

**********

4. Have the authors made all data underlying the findings in their manuscript fully available?

The PLOS Data policy

Reviewer #1: Yes

**********

5. Is the manuscript presented in an intelligible fashion and written in standard English?

Reviewer #1: Yes

**********

Reviewer #1: I thank the authors for their thoughtful revisions and for addressing my concerns. I am pleased to see the effort invested in generating data that allow synchrony to be studied in a meaningful contextual setting. The manuscript offers a strong and much-needed contribution, and I hope this study will encourage researchers to design more work that directly examines synchrony in context.

**********

what does this mean?). If published, this will include your full peer review and any attached files.

If you choose “no”, your identity will remain anonymous but your review may still be made public.

**Do you want your identity to be public for this peer review?**  For information about this choice, including consent withdrawal, please see our Privacy Policy

Reviewer #1: **Yes:**  Alon Burns

**********

# Editor report: Acceptance letter

**DOI:** 10.1371/journal.pone.0356071.r008

**Authors:** Sebastian Wallot

PONE-D-25-41087R3

PLOS One

Dear Dr. Fora,

I'm pleased to inform you that your manuscript has been deemed suitable for publication in PLOS One. Congratulations! Your manuscript is now being handed over to our production team.

At this stage, our production department will prepare your paper for publication. This includes ensuring the following:

* All references, tables, and figures are properly cited

* All relevant supporting information is included in the manuscript submission,

* There are no issues that prevent the paper from being properly typeset

You will receive further instructions from the production team, including instructions on how to review your proof when it is ready. Please keep in mind that we are working through a large volume of accepted articles, so please give us a few days to review your paper and let you know the next and final steps.

Lastly, if your institution or institutions have a press office, please let them know about your upcoming paper now to help maximize its impact. If they'll be preparing press materials, please inform our press team within the next 48 hours. Your manuscript will remain under strict press embargo until 2 pm Eastern Time on the date of publication. For more information, please contact onepress@plos.org.

You will receive an invoice from PLOS for your publication fee after your manuscript has reached the completed accept phase. If you receive an email requesting payment before acceptance or for any other service, this may be a phishing scheme. Learn how to identify phishing emails and protect your accounts at https://explore.plos.org/phishing.

If we can help with anything else, please email us at customercare@plos.org.

Thank you for submitting your work to PLOS ONE and supporting open access.

Kind regards,

PLOS ONE Editorial Office Staff

on behalf of

Prof. Sebastian Wallot

Academic Editor

PLOS One
